# Magnetic Nanoparticles with On-Site Azide and Alkyne Functionalized Polymer Coating in a Single Step through a Solvothermal Process

**DOI:** 10.3390/pharmaceutics16091226

**Published:** 2024-09-19

**Authors:** Romualdo Mora-Cabello, David Fuentes-Ríos, Lidia Gago, Laura Cabeza, Ana Moscoso, Consolación Melguizo, José Prados, Francisco Sarabia, Juan Manuel López-Romero

**Affiliations:** 1 Department of Organic Chemistry, Faculty of Sciences, University of Málaga, 29071 Málaga, Spain; romumora28a@gmail.com (R.M.-C.); davidfuentesrios@uma.es (D.F.-R.); anaisabelmoscosoreina@gmail.com (A.M.); frsarabia@uma.es (F.S.); 2 Institute of Biopathology and Regenerative Medicine (IBIMER), Biomedical Research Center (CIBM), 18100 Granada, Spain; lgago@ugr.es (L.G.); melguizo@ugr.es (C.M.); jcprados@ugr.es (J.P.); 3 Instituto de Investigación Biosanitaria de Granada (Ibs.GRANADA), 18012 Granada, Spain; 4 Department of Anatomy and Embryology, University of Granada, 18071 Granada, Spain

**Keywords:** CBD-derivatives, magnetite, metalloorganic nanoparticles, surface functionalization, hyperthermia, SPION

## Abstract

**Background/Objectives**: Magnetic Fe_3_O_4_ nanoparticles (MNPs) are becoming more important every day. We prepared MNPs in a simple one-step reaction by following the solvothermal method, assisted by azide and alkyne functionalized polyethylene glycol (PEG400) polymers, as well as by PEG6000 and the polyol β-cyclodextrin (βCD), which played a crucial role as electrostatic stabilizers, providing polymeric/polyol coatings around the magnetic cores. **Methods**: The composition, morphology, and magnetic properties of the nanospheres were analyzed using Transmission Electron and Atomic Force Microscopies (TEM, AFM), Nuclear Magnetic Resonance (NMR), X-ray Diffraction Diffractometry (XRD), Fourier-Transform Infrared Spectroscopy (FT-IR), Matrix-Assisted Laser Desorption/Ionization (MALDI) and Vibrating Sample Magnetometry (VSM). **Results**: The obtained nanoparticles (@Fe_3_O_4_-PEGs and @Fe_3_O_4_-βCD) showed diameters between 90 and 250 nm, depending on the polymer used and the Fe_3_O_4_·6H_2_O precursor concentration, typically, 0.13 M at 200 °C and 24 h of reaction. MNPs exhibited superparamagnetism with high saturation mass magnetization at room temperature, reaching values of 59.9 emu/g (@Fe_3_O_4_-PEG6000), and no ferromagnetism. Likewise, they showed temperature elevation after applying an alternating magnetic field (AMF), obtaining Specific Absorption Rate (SAR) values of up to 51.87 ± 2.23 W/g for @Fe_3_O_4_-PEG6000. Additionally, the formed systems are susceptible to click chemistry, as was demonstrated in the case of the cannabidiol-propargyl derivative (CBD-Pro), which was synthesized and covalently attached to the azide functionalized surface of @Fe_3_O_4_-PEG400-N_3_. Prepared MNPs are highly dispersible in water, PBS, and citrate buffer, remaining in suspension for over 2 weeks, and non-toxic in the T84 human colon cancer cell line, **Conclusions**: indicating that they are ideal candidates for biomedical applications.

## 1. Introduction

It has been theorized that the emergence of novel properties and potential applications in materials with tiny and monodispersed dimensions, would constitute a significant advancement [1]. Therefore, the development and synthesis of magnetic nanoparticles (MNPs) has gained crucial relevance in research, generating a continuously growing interest due to their potential applications in areas such as chemistry, physics, or electronics [2]. Medical applications have also been strongly developed, ranging from magnetic resonance imaging and medical diagnostics to magnetic hyperthermia and drug delivery technologies [3,4,5].

The main limitation of MNPs is their inherent long-term instability. Degradation occurs through two main pathways: (1) loss of dispersibility, where MNPs tend to aggregate due to Van der Waals forces, overcoming the high surface energy and strong magnetic attraction between particles; and (2) loss of magnetism, when oxidation of MNPs occurs [2,6]. These issues can be addressed by developing a functionalized surface around the MNP cores, providing a protective shell where it can be covalently attached to different species, such small molecules, peptides, or even antibodies [7,8]. Moreover, this functionalization can improve biocompatibility and hydrophilicity of the magnetic material [8]. In the case of superparamagnetic systems, the response to an external magnetic field is even better, improving individual properties and their application through magnetic hyperthermia therapies [9].

Several studies have shown that the encapsulation of MNPs prevents agglomeration by reducing available surface area, and thus indirectly decreasing surface energy which conduces agglomeration [10,11,12,13]. Encapsulation also creates a protective layer around naked MNPs, preventing them from oxidizing to γ-Fe_2_O_3_ after prolonged exposure to air [14]. The introduction of this coating layer onto the surface of MNPs ensures the chemical stability of MNPs and preserves their magnetic properties. Coated MNPs also present advantages in biomedical applications compared to naked MNPs, including lower cytotoxicity, higher cytocompatibility, and better bioconjugation capability due to the presence of reactive materials on its surface [15], which, together with its magnetic potential, could be useful for the treatment of certain diseases such as cancer. In order to address this point, it is necessary to functionalize and coat MNPs with specific polymers and macromolecules designed to improve their conjugation. This involves a precise sequence involving, first, the synthesis of MNPs, second, surface functionalization, and third, polymerization and coating of the magnetic cores, all within a relatively short time frame to prevent agglomeration [10,11,12,13].

One of the most distinguished methods to synthesize magnetite is solvothermal decomposition, initially reported by Deng et al., which prepares monodispersed single-crystal ferrite microspheres, affording a size distribution of 90–200 nm [16]. Many studies have applied this methodology to prepare MNPs. The search for different size ranges controlling the reaction time [17], the analysis of the effect of surfactants [18], the use of urea as a homogeneous precipitant [19], or citrate groups as electrostatic stabilizers [20,21], have been studied. PEG coating has also been carried out for the stabilization of magnetite nanoparticles, together with the common use of polyols with various reducing capacities, for MNP preparation [9,22,23,24]. βCD-Citrate and carboxymethyl-βCD derivatives have also been used for stabilization of gum-arabic-modified magnetite and pristine magnetite nanosystems, respectively, via carbodiimide activation [25,26]. More complex derivatives, such as mono-6-deoxy-6-(*p*-tolylsulfonyl)-βCD, have been used to surface modify and stabilize Fe_3_O_4_ nanoparticles using electrochemical methods [27]. More recently, βCD combined with ionic liquids was used for coating of co-precipitation prepared MNPs [28], while copolymers as βCD-co-(methacryloyloxy)ethyl phosphorylcholine were used for coating Fe_3_O_4_ nanoparticles in hemocompatibility studies [29]. All these methods involve the synthesis of MNPs prior to surface functionalization.

However, to the best of our knowledge, minimal research has been conducted regarding the synthesis of MNPs using previously functionalized PEGs. Here we present our results on the preparation of MNPs by following the well-known solvothermal method, but using functionalized polymers as precursors, specifically, PEG400 and its alkyne and azide derivatives, which act as surfactants and stabilizers. Additionally, PEG6000 and the polyol βCD are also tested. The preparation is carried out in a one-step process that results in obtaining MNPs with a functionalized, hydrophilic, and protective layer over the magnetic clusters. In this sense, we expand the study by analyzing the magnetic properties, hyperthermia behavior, and biocompatibility of the nanoformulations in vitro. Moreover, the mentioned functional groups enable the performance of click chemistry reactions, which represents a substantial contribution due to the versatility and potential that these reactions offer [30]. To illustrate this point, we carried out the preparation of a CBD-alkyne derivative to be incorporated using covalent bond formation via the click reaction to the azide-modified surface of the MNP. This approach marks a significant advance toward creating multifunctional and versatile systems that can be adapted for a wide range of biomedical applications, including targeted drug delivery.

## 2. Materials and Methods

### 2.1. Chemicals

Iron(III) chloride hexahydrate, *N*,*N*-dimethylformamide (DMF), NaN_3_, propargyl chloride, propargyl bromide, dichloromethane (DCM), 3-bromopropionyl chloride, ethanol, PEG400, PEG6000, ethylene glycol, sodium acetate, β-cyclodextrin, iron(III) acetylacetonate, and triethylamine (TEA) were purchased from Merck. All chemicals were used without further purification. Cannabidiol (CBD) was synthesized according to reference [31].

### 2.2. Characterization Techniques

The morphology of the MNPs were determined using a JEOL JEM 1400 Transmission Electron Microscope (TEM). Samples consisted of dispersed nanoparticles in ethanol or water onto copper grids with a carbon substrate. After allowing the nanoparticles to dry on the grid, they were visualized for analysis. Atomic Force Microscopy (AFM) was also used for the same purpose, focusing on studying the topography and interactions of the ELR (Electrostatic-Langmuir-Rodin) with molecules. Images were obtained using the AFM equipment (diMultiModeTMV from Veeco Instruments—Nanoscope V, Madrid, Spain) and the tapping technique. During measurement, a resolution of 256 lines/area was used, and scanning was performed over an area of 10 μm × 10 μm.

The chemical structure of the MNPs was characterized using an X-ray Diffractometer (XRD). Transmission powder X-ray diffraction data using MoKα1 radiation (0.071093 nm) was recorded using a Bruker D8 ADVANCE diffractometer. The incident beam consists of a primary monochromator + focusing mirror, a 2 mm anti-scatter slit, and a 2.5° Soller were used in both the incident and transmitted beams. The detection system consists of an EIGER detector (from the commercial company DECTRIS) specially designed and optimized for Mo anodes. The detector was used with an aperture of 4 × 21°, working in VDO mode. Measurements were made from 2 to 80° (2θ) within a period of 120 min. The tube worked at 50 kV and 50 mA. Functional groups and bonds within the MNPs were examined using a Bruker Vertex70 Fourier-Transform Infrared Spectrometer (FT-IR, Bruker, Madrid, Spain). Measurements were conducted using attenuated total reflectance with the Golden Gate Single Reflection Diamond ATR System accessory, directly exposing the sample to air without the need for dispersion or treatment. Spectra acquisition employed a standard spectral resolution of 4 cm^−1^ in the spectral range of 4000–400 cm^−1^ with 64 accumulations. Additionally, Nuclear Magnetic Resonance (NMR) spectra were obtained using a Bruker Advanced III 500 instrument. ^1^H spectra were recorded at 500 MHz, and ^13^C spectra at 100 MHz, respectively. All compounds were dissolved in deuterated chloroform (CDCl_3_) or deuterated dimethyl sulfoxide (DMSO-d6). Shift values are expressed in ppm, referenced to TMS. For molecular mass distributions a MALDI-TOF/TOF UltrafleXtreme (Bruker Daltonics, Madrid, Spain) was used. 2,5-Dihydroxybenzoic acid (DHB) or α-cyano-4-hydroxy-cynamic acid (CHCA) were used as matrixes. All spectra were obtained in the positive ion mode using an accelerating voltage of 8 kV for the first source, 15 kV for the second source, with a laser intensity of ~10% greater than threshold. Samples used for the MALDI analyses were prepared as follows: 10 mL of MNP solution (3–4 mg/mL in CHCl_3_) were mixed with 30 mL of DHB or CHCA solution (0.1 M in CHCl_3_/THF 90/10 *v*/*v*). This solution was added to 1 mL of a 0.01 M solution of CF_3_COONa salt as a cationizating agent in THF as solvent. Then 1 mL of each analyte/matrix/salt mixture was spotted on the MALDI sample holder and slowly dried to allow analyte/matrix co-crystallization.

The analysis of the magnetization behavior was performed on a Vibrating Sample Magnetometer (8600 Series VSM, Lake Shore Cryotronics, Columbus, OH, USA) using magnetic fields ranging from −19,000 to 19,000 Oe at 300 K, with sample concentrations of 1 g/L of Fe (100 µL) dropped over a cotton ball. The diamagnetic component of the cotton was subtracted as background.

### 2.3. Specific Absorption Rate Analysis

SAR was calculated to determine the magnetic properties and ability to generate hyperthermia in nanoformulations. Commercial equipment (D5 Series from nB nanoScale Biomagnetics, Pittsburgh, PA, USA) and a fiber optic sensor were used to measure temperature rise. All nanoformulations were diluted at 0.5 mg/mL Fe concentration in 0.5 mL of water. They were subjected to an alternating magnetic field (385 kHz, 28 kA/m) for 25 min. The SAR value (W/g) was calculated using Equation (1) where *C*pH_2_O was the specific heat of water (J/g K), *m*H_2_O was the mass of water (g), *m*BMLs was the mass of Fe (g) present in all nanoformulations and (d*T*/d*t*) was the slope of temperature rise within the first 1500 s of the heating curve [32]:

(1) $$SAR=\frac{C_{p}H_{2}O\times mH_{2}O}{mBMLs}\times \frac{dT}{dt}\left[{Wg_{Fe}}^{-1}\right]$$

### 2.4. Synthesis of PEG Derivatives: PEG400-N_3_ and PEG400-Pro (Scheme 1)

PEG400-N_3_: In a 250 mL flask with Ar inlet, an amount of PEG400 (3.54 mL) was dissolved in DCM (90 mL) at −5 °C. To this solution, 3-bromopropanoyl chloride (502 μL, 0.854 g, 0.005 mol) in DCM (20 mL) was added throughout a period of 20 min by using a programmable syringe pump. The solution was stirred for 30 min at −5 °C. After this period, the reaction mixture was left to reach room temperature (21 °C). Then the solvent was removed in vacuum to produce a yellowish syrup. Under Ar, PEG-Br (4.73 g), H_2_O or DMF (90 mL), and NaN_3_ (0.45 g, 7 mmol) were subsequently added to this syrup. The flask containing the reaction mixture was heated at 70 °C (silicone bath) and magnetically stirred for 12 h. After this period, the residue was drained into diethyl ether (200 mL) to separate the PEG400-N_3_. Organic mixture was then washed with brine (3 × 15 mL), dried over anhydrous MgSO_4_, and filtered off. Filtrates were concentrated to dryness under vacuum to produce PEG400-N_3_ as a colorless syrup (3.94 g, 90% yield).

**Scheme 1:**
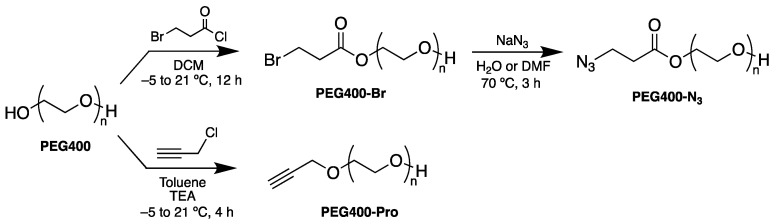
Two-step synthesis of PEG400-N_3_ and synthesis of PEG400-Pro.

PEG400-Pro: under Ar, in a 100 mL round bottom flask, PEG400 (0.885 mL) and TEA (3 drops) were mixed with anhydrous toluene (20 mL). The flask was cooled at −5 °C. To this mixture, a solution of propargyl chloride (10 μL, 0.01 mmol) in anhydrous toluene (20 mL) was added dropwise over a period of 20 min, using the programmable syringe pump. The reaction mixture was stirred for 60 min at −5 °C. After this period, the mixture was left to warm up to room temperature (21 °C) over a period of about 4 h. Then the reaction mixture was washed in brine (3 × 5 mL), dried over anhydrous MgSO_4_, and filtered off. Finally, filtrates were concentrated to dryness under vacuum to obtain PEG400-Pro as a colorless syrup (978 mg, 98 % yield).

NMR, FT-IR, and MALDI Spectra for these compounds are included in Supplementary Materials (Figures S1–S11).

### 2.5. Synthesis of MNPs

In a typical procedure, FeCl_3_·6H_2_O (1.35 g, 5 mmol) was dissolved in ethylene glycol (40 mL) to form a clear solution, followed by the addition of NaAcO (3.6 g, 44 mmol) and PEG or derivatives (PEG400, PEG6000, PEG400-N_3_, PEG400-Pro) or βCD (1.0 g). The mixture was stirred vigorously at 21 °C for 30 min and then transferred into sealed tubes. These tubes were heated at 200 °C for 24 h. After this period, the reaction mixture was allowed to cool at 21 °C. The black solid obtained was decanted, washed three times with ethanol, then dried at 70 °C for 3 h to produce @Fe_3_O_4_-PEG400, @Fe_3_O_4_-PEG6000, @Fe_3_O_4_-PEG400-N_3_, @Fe_3_O_4_-PEG400-Pro, and @Fe_3_O_4_-βCD MNPs (Table 1) systems, which were finally dispersed in ethanol (20 mL, Table 1).

### 2.6. Synthesis of CBD-Pro Derivatives and @Fe_3_O_4_-PEG400-N_3_ Covalent Surface Functionalization with CBD to Obtain @Fe_3_O_4_-PEG400-CBD (Scheme 2)

CBD-Pro-1 and CBD-Pro-2: CBD (200 mg, 0.64 mmol) was dissolved in DMF (6.5 mL). The solution was cooled to 0 °C and then propargyl bromide (0.274 mL, 3.2 mmol) and K_2_CO_3_ (131.6 mg, 0.95 mmol) were added. This suspension was stirred at room temperature for 48 h. Upon completion of the reaction, the reaction solution was extracted with ethyl acetate (3 × 300 mL). The organic phase was then dried with anhydrous Mg_2_SO_4_, and the solvent was removed under vacuum to produce a crude product, which was further purified by column chromatography (petroleum ether/ethyl acetate, 90:1) to give CBD-2′-propynyl ether (CBD-Pro-1) and CBD-2′,6′-dipropynyl ether (CBD-Pro-2) [33]. CBD-Pro-1: 50% yield, ^1^H-NMR (CDCl_3_, 500 MHz) δ(ppm): 6.34 (s, 1H), 6.29 (s, 1H), 6.03 (s, 1H), 5.57 (s, 1H), 4.58−4.53 (m,2H), 4.31 (s, 1H), 4.00 (d, J = 10.1 Hz, 1H), 2.50−2.47 (m, 2H), 1.68 (s, 3H), 0.88 (t, J = 7.0 Hz, 3H). CBD-Pro-2: 45% yield, ^1^H-NMR (CDCl_3_, 500 MHz) δ(ppm): 6.45 (s, 2H), 5.21 (s, 1H), 4.62−4.55 (m, 4H), 4.43 (dd, J = 2.6 Hz, 2H), 4.00−3.97 (m, 1H), 2.88 (td, J = 10.8 Hz, 1H), 2.54 (t, J = Hz, 2H), 2.45 (t, 2H), 1.97 (d, J = 17.7 Hz, 1H), 1.79−1.70 (m, 2H), 1.61−1.56 (m, 2H), 1.38−1.28 (m, 4H), 0.89 (t, J = 6.9 Hz, 3H). See Supplementary Materials Figures S12–S14.

**Scheme 2:**
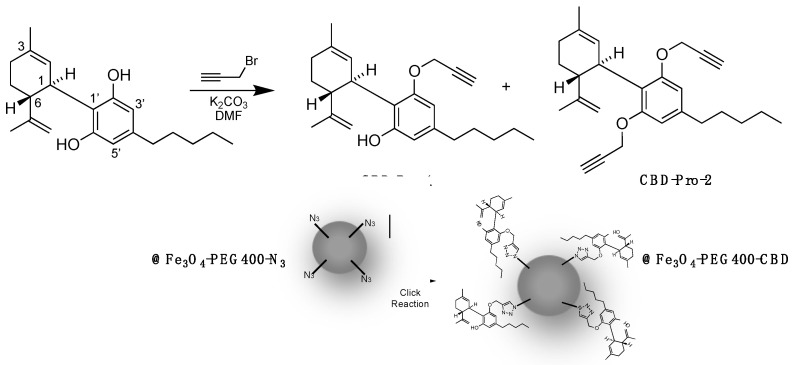
Synthesis of propargyl derivatives of CBD and preparation of @Fe_3_O_4_-PEG400-CBD.

@Fe_3_O_4_-PEG400-CBD: in a reaction vial containing CBD-Pro-1, a solution (5 mL) of CuSO_4_ (2.5 mM), and sodium ascorbate (50 mM), water was added. After 5 min, a solution of @Fe_3_O_4_-PEG400-N_3_ (10 mM) in degassed methanol/water (1:1 *v*/*v*) was added. After incubation for 4 h under Ar, the reaction mixture was diluted with water (20 mL) and extracted with DCM (2 × 5 mL). Organic extracts were dried over anhydrous MgSO_4_, filtered off, and concentrated to dryness under vacuum to give @Fe_3_O_4_-PEG400-CBD samples, which were analyzed by FT-IR (Supplementary Materials Figure S15).

### 2.7. In Vitro Proliferation Assay

The human colon cancer cell line T84 was obtained from the American Type Culture Collection (Rockville, MD, USA). Cells were grown in Dulbecco’s Modified Eagle’s Medium (DMEM), and supplemented with 10% fetal bovine serum (FBS) and 1% of penicillin-streptomycin (Sigma–Aldrich, Madrid, Spain). The cell line was maintained at 37 °C in an atmosphere containing 5% CO_2_. Cells were seeded in 48-well plates at a concentration of 5 × 10^3^ cells/well and were incubated overnight. MNPs coated with PEG400, PEG6000, and βCD were then administered at a concentration of Fe_3_O_4_ ranging from 1 to 100 µg/mL and were incubated for 72 h. Sulforhodamine B (SRB) assay was used to determine the relative proliferation percentage and the optical density of the samples was measured at 492 nm (Titertekmultiscan Colorimeter, Flow, Irvine, CA, USA), as described previously [34].

## 3. Results and Discussion

### 3.1. Synthesis of PEG Derivatives

The PEG400 was synthetically modified to incorporate alkyne through the propargyl moiety (PEG400-Pro) or azide (PEG400-N_3_) groups, and then used for the preparation of the MNPs. The synthesis of PEG400-N_3_ was carried out in a two-step process using PEG400 as a starting material. First, PEG400-Br was prepared in good yield by treating PEG400 with 3-bromopropanoyl chloride using DCM as solvent, and second, PEG400-Br was made to react with sodium azide to afford the PEG400-N_3_ derivative. Finally, the reaction was tested using anhydrous DMF and H_2_O as solvents, reaching similar yields in both cases, 82.5% and 78.2%, respectively. Therefore, subsequent repetitions of the synthesis were performed in H_2_O due to cost savings and speed of synthesis, as it was not necessary to set up the distillation apparatus to obtain anhydrous DMF. Synthesis of PEG400-Pro was conducted by treating PEG400 with propargyl chloride in dry toluene in the presence of TEA, and under moisture-free conditions. We observed that, in the presence of traces of water, the formation of the product does not occur.

The obtention of the PEG400-Br, PEG400-N_3_ and PEG400-Pro systems was confirmed by NMR, FT-IR, and MALDI (Supplementary Materials Figures S1–S11). The formation of the PEG400-Br was confirmed by the ^1^H-NMR spectrum, which shows the presence of a signal corresponding to the methylene group attached to the bromine atom at 4.1 ppm, integral 2H, and another signal at 2.8 ppm (2H) assigned to the methylene moiety vicinal to the carbonyl group. The presence of C = O was also confirmed by the quaternary carbon characteristic peak at 170.3 ppm in the ^13^C-NMR and the intense IR absorption at 1600 cm^−1^. For PEG400-N_3_, in the ^1^H-NMR, the peaks corresponding to one methylene attached to an azide group at 2.6 ppm (2H) and these assigned to a carbonyl group at 4.2 ppm (2H) are observed, where the methylene chemical shift differs from PEG400-Br. The characteristic peak of the carbonyl carbon is also visible (171.4 ppm, ^13^C-NMR). FT-IR abortion at 2102 cm^−1^ confirms the presence of the azide group. In the case of PEG400-Pro, the formation of the alkyne terminal group was confirmed by the characteristic signal at 3.0 ppm (1H) in the ^1^H-NMR, which can be assigned to the alkyne terminal hydrogen. The presence of the alkyne moiety was also confirmed by the infrared analysis (FT-IR), in which was clearly observed a highly intense bending vibration frequency at 737 cm^−1^ assigned to the triple bond group. MALDI-TOF analyses were also carried out. Analysis of PEG400 standard shows that, with the addition of Na^+^, a single series of PEG400 ions completely dominate the spectrum. The [H(C_2_H_4_O)_n_OH + Na]^+^ ions at m/z 365, 409, 453, 497, 541, 585, 629, 673, 717, and 761 correspond to PEG400 oligomers with the number of repeating units being n = 8–17. In the case of PEG400-N_3_, two characteristics of ions can be observed at about m/z 613 and 657, which are attributed to the substitution of the hydroxyl group by the azide group, and correspond to the mass increase of m/z of 28 with respect to the m/z 585 and 629 in PEG400. Similarly, the substitution of a hydroxyl group was confirmed by the peak of m/z 481, representing an increase of about 30 mass units with respect of the peak of m/z of 453 in PEG400, due to the incorporation of the propargyl moiety.

### 3.2. Preparation of MNPs Systems

Typical syntheses of Fe_3_O_4_ using the co-precipitation or oxidation methods require the addition of cationic surfactants, such as CTAB, for the stabilization and monodispersity of MNPs [11]. It is known that MNPs exhibit a pronounced tendency to agglomerate during their formation in the liquid-phase process [35]. Additionally, for the polymerization with certain polymers like pNIPAM or p4VP, prior functionalization of the magnetic cores is necessary to enable the binding of specific molecules [36]. Conversely, for polymers like PEG, prior functionalization of the magnetic cores is not required due to the inherent chemical and physical affinity between PEG and the surface of MNPs [11]. The hydroxyl groups of PEG can interact through hydrogen bonding and London dispersion forces with the surface groups of magnetite.

To achieve all in situ monodispersed and coated Fe_3_O_4_ nanoparticles, we followed the synthetic procedure of the solvothermal method, involving three critical additions. First, NaAcO was added for electrostatic stabilization and to prevent particle agglomeration. In our system, the presence of NaAcO was crucial as it seemed to facilitate the reduction of FeCl_3_ to Fe_3_O_4_ using ethylene glycol. Second, PEG and the different derivatives that we have synthesized were added, in order to act as surfactants or stabilizers. PEG inherently provided a hydrophilic coating to the MNPs, contributing to preventing particle agglomeration. The third critical feature was the increase of the reaction temperature up to 200 °C, an indispensable requirement for producing Fe_3_O_4_.

The solvothermal reduction reaction was carried out by heating the solution at 200 °C and maintaining this temperature for 24 h within sealed flasks (Table 1). The heat provided during the process promotes the reduction reaction, allowing metallic ions to transform into magnetic nanoparticles. In contrast to the thermal decomposition method which produces hydrophobic MNPs in an organic phase [36], this method generates water-dispersible nanoparticles through a thermal decomposition process in hydrophilic polyalcoholic solvents. Polyalcohols serve three functions: a high boiling point solvent, a reducing agent, and a stabilizer to control nanoparticle growth and prevent aggregation. The use of polyols as a surfactant or stabilizer results in the formation of MNPs with an inherent hydrophilic coating. This simplifies the use of these nanoparticles for subsequent biological applications, as no modification is needed to enhance their dispersion in water, and both the synthesis of the magnetite cores and the polymeric coating occur in a single step.

**Figure 1:**
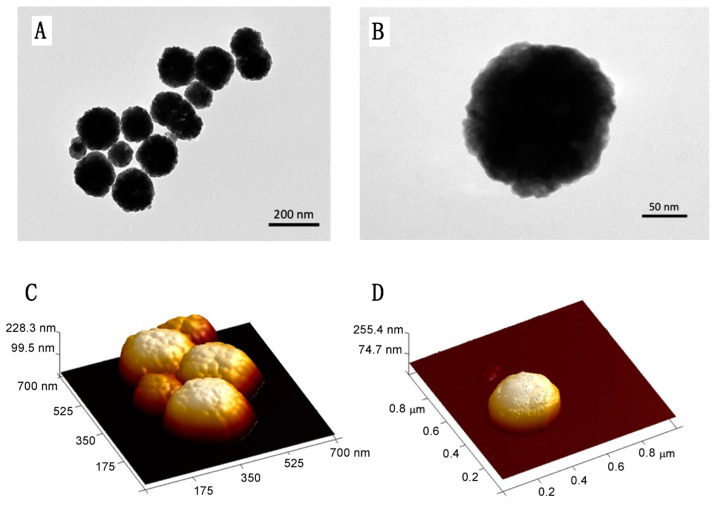
The microscopy study results: (**A**) TEM image of @Fe_3_O_4_-PEG400 nanospheres, (**B**) TEM image of a single @Fe_3_O_4_-PEG400 nanosphere, (**C**) AFM image of @Fe_3_O_4_-PEG400, and (**D**) AFM image of @Fe_3_O_4_-PEG400 single nanosphere.

The size and shape of the prepared MNPs were examined using TEM and AFM. Figure 1 shows representative images of the MNPs’ structures prepared through the solvothermal reduction method. Well-dispersed spherical clusters containing aggregates of Fe_3_O_4_ NP, with very narrow diameter distributions, are observed. Furthermore, the shape and size of the Fe_3_O_4_ products with different polymeric coatings remained unchanged in morphology compared to those of the Fe_3_O_4_ precursor with PEG400.

**Figure 2:**
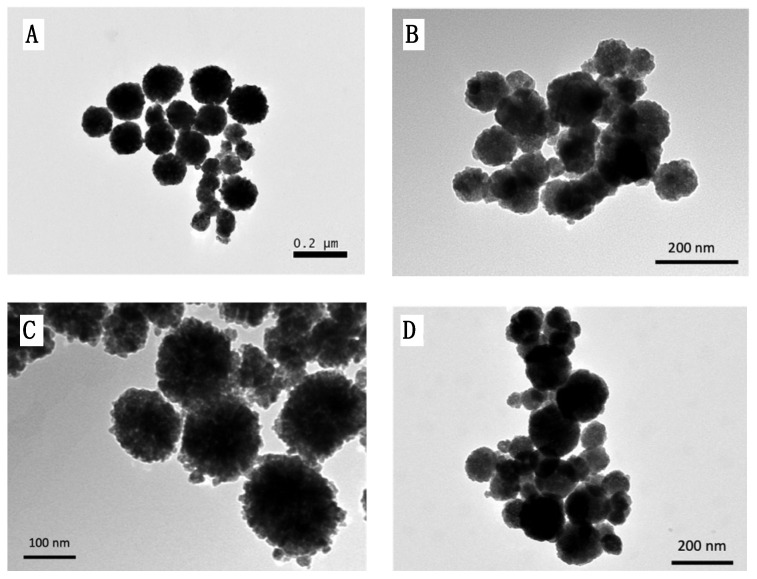
TEM images of: (**A**) @Fe_3_O_4_-PEG400-Pro, (**B**) @Fe_3_O_4_-PEG400-N_3_, (**C**) @Fe_3_O_4_-βCD, and (**D**) @Fe_3_O_4_-PEG6000.

The diameters of the MNPs were influenced by the type of polymeric coating (Table 1). Under the same reaction conditions, with the Fe_3_O_4_·6H_2_O precursor concentration of 0.13 M, a temperature of 200 °C, and a reaction time of 24 h, a diameter of approximately 250 nm was obtained for @Fe_3_O_4_-PEG400. Meanwhile, 90 nm was measured for @Fe_3_O_4_-PEG400-N_3_, measuring 150 nm for @Fe_3_O_4_-PEG6000, 155 nm for @Fe_3_O_4_-βCD, and 200 nm for @Fe_3_O_4_-PEG400-Pro (Figure 2, ImageJ Software, https://imagej.net/). In all cases the diameter for the magnetite cores was about 10 nm.

**Figure 3:**
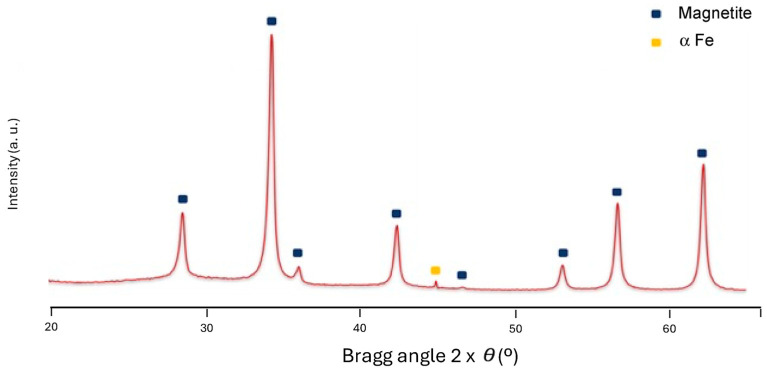
Representative XRD diffractogram of Fe_3_O_4_ nanoparticles coated with PEG (@Fe_3_O_4_-PEG6000).

**Figure 4:**
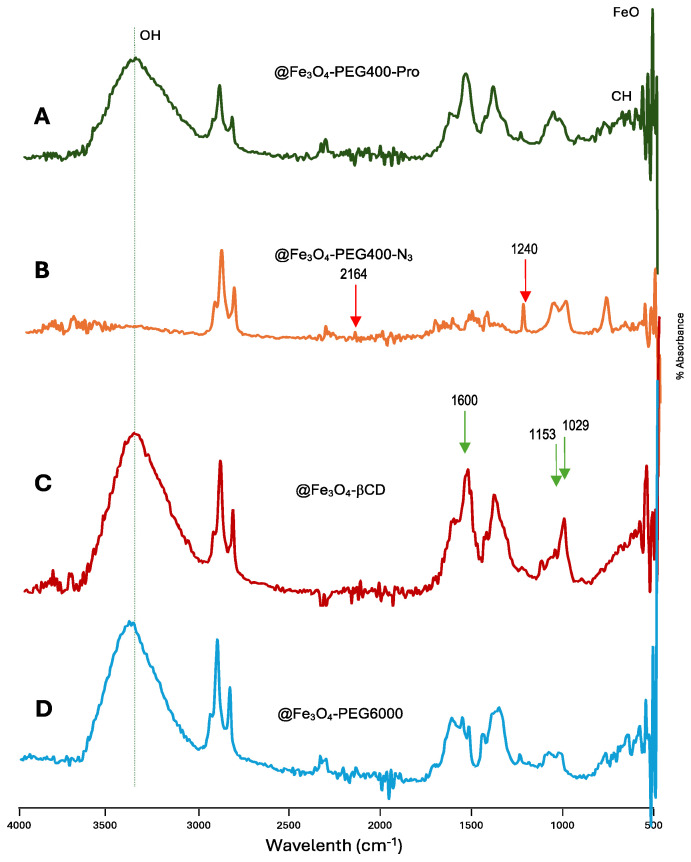
FT-IR Spectra of: (**A**) @Fe_3_O_4_-PEG400-Pro, (**B**) @Fe_3_O_4_-PEG400-N_3_, (**C**) @Fe_3_O_4_-βCD, and (**D**) @Fe_3_O_4_-PEG6000 samples.

The chemical composition of MNPs was studied by XRD and FT-IR techniques (Figure 3 and Figure 4, respectively). Samples of Fe_3_O_4_ were analyzed by XRD, an effective technique to characterize the crystalline structure of synthesized magnetic nanoparticles. As the polymer and polyol are poorly crystalline material, they are not seen in XRD, and only the magnetite can be observed. Figure 3 shows the XRD pattern of @Fe_3_O_4_-PEG6000, representative for the prepared systems, showing the six magnetite characteristics peaks at 2θ of 28.7° (2 2 0), 34.8° (3 1 1), 43.5° (4 0 0), 53.8° (4 2 2), 57.5° (5 1 1), and 62.4° (4 4 0). The XRD pattern matches well with the standard XRD data for bulk magnetite [16], with almost no impurity phase (α-Fe). Additionally, a lattice parameter was obtained from the XRD pattern, being nearly 8.5 Å, and indicating a Fe_3_O_4_ material for the @Fe_3_O_4_-PEG6000 systems [37].

Moreover, the Scherrer equation was used for the calculation of particle sizes for this sample (@Fe_3_O_4_-PEG6000):

(2) $$D=\frac{K\lambda }{\beta \cos2\theta }$$

where K is the Scherrer constant, *λ* is wavelength of the X-ray beam used, *β* is the full width at half maximum (FWHM) of the peak, and *θ* is the Bragg angle [38]. The Scherrer constant denotes the shape of the particle, and its value was taken as 0.89 for spherical nanoparticles. The diameter of the MNPs was estimated at 8.7 ± 0.4 nm as an average of the FWHM of the six magnetite characteristics’ peaks. This value is in concordance with that obtained from TEM micrographs of about 10 nm (Figure 2).

Samples @Fe_3_O_4_-PEG400-Pro, @Fe_3_O_4_-PEG400-N_3_, @Fe_3_O_4_-βCD, and @Fe_3_O_4_-PEG6000 were chemically characterized by FT-IR to identify the coating on the surface of the nanoparticles (Figure 4). FT-IR provides evidence for the existence of the different functional groups in the coating.

Figure 4A displays the spectrum for @Fe_3_O_4_-PEG400-Pro, where the bending deformation band of acetylene can be seen at 608 cm^−1^. In Figure 4B, corresponding to @Fe_3_O_4_-PEG400-N_3_, the azide absorption band is observed at frequencies of 1240 and 2164 cm^−1^ (red arrows). Additionally, in the four IR spectra, the vibration (560–590 cm^−1^) of the Fe-O bond in reference to that of the magnetite present is observed. The pure magnetite systems exhibited a broad absorption peak of around 3200 cm^−1^, which was assigned to the OH stretching vibration from surface hydroxyl groups. This is also one of the most intense bands in @Fe_3_O_4_-PEG400-βCD (Figure 4C), where it corresponds to the multiple OH functional groups present, but also to adsorbed water. It is worth noting that OH stretching is almost not detected in the @Fe_3_O_4_-PEG400-N_3_ spectrum (Figure 4B), corresponding to a mostly complete surface substitution of OH by azide groups. On the other hand, the bands assigned to the vibrations of the methylene chains, C–O, and C–C groups are observed in the interval of 840–1500 cm^−1^. As expected, the most intense βCD vibrations are observed at 1029, 1153, and 1600 cm^−1^ in the case of the @Fe_3_O_4_-βCD sample (Figure 4C, green arrows).

### 3.3. Preparation of @Fe_3_O_4_-PEG400-CBD

As a proof of concept, we carried out the surface functionalization of @Fe_3_O_4_-PEG400-N_3_ using treatment with CBD-Pro-1 (Scheme 2). After 4 h of reaction time, the formation of the @Fe_3_O_4_-PEG400-CBD system was confirmed by FT-IR, where the characteristic bands of the triazol moiety were observed at 1485, 1529, and 1547 cm^−1^ (Supplementary Materials Figure S15)

### 3.4. Magnetic Measurements

The magnetic properties of the ferrite nanospheres were investigated using a VSM. Superparamagnetism is defined as the reaction to an applied magnetic field without retaining any magnetization after its removal. It denotes re-dispersion of the magnetic nanoparticles in solution without the occurrence of severe aggregation from which ferromagnetic nanoparticles often suffer, which limits their applications. The superparamagnetic behavior of MNPs is highly desirable due to their high efficiency when absorbing the energy of an alternating magnetic field and converting it into heat [39].

**Figure 5:**
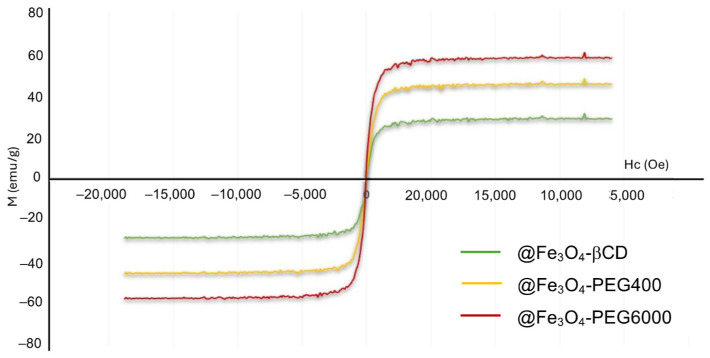
Magnetization curves of @Fe_3_O_4_ coated with βCD, PEG400, or PEG6000.

Figure 5 shows magnetization curves measured at 300 K for Fe_3_O_4_ nanospheres coated with the PEG400, PEG6000, and βCD (@Fe_3_O_4_-PEG400, @Fe_3_O_4_-PEG6000, and @Fe_3_O_4_-βCD samples). Qualitative analysis of the hysteresis curves, where no remanence and coercivity values are observed in the magnetization, indicated that the MNPs are superparamagnetic, usually named as SPION (Superparamagnetic Iron Oxide Nanoparticles), with no observed ferromagnetism. Furthermore, the MNPs were well dispersed in water and could be separated from the solution using the attraction of a magnet.

The measured saturation mass magnetization values for the prepared MNPs were found to be 59.9 emu/g for @Fe_3_O_4_-PEG6000, 47.8 emu/g for @Fe_3_O_4_-PEG400, and 30.8 emu/g for @Fe_3_O_4_-βCD. Among them, the coated βCD nanoparticles showed lower saturation mass magnetization value than that for PEG-coated nanoparticles, and all of them lower than non-coated MNPs. This fact is known, and it is attributed to the non-magnetic material covering the surface of the Fe_3_O_4_ nanoparticles, which confirms the presence of organic molecules coating the surface. As can be seen (Figure 5), magnetization curves do not show any histeresis, meaning coercive force values of 0 or close to 0. This fact is in concordance with SPM nanoparticles with sizes up to 10 nm [40].

### 3.5. Hyperthermia and In Vitro Proliferation Analyses

**Figure 6:**
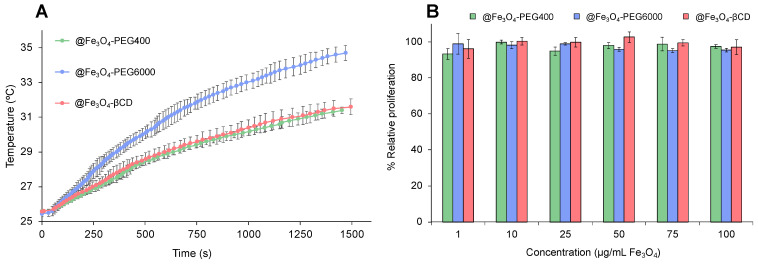
Hyperthermia and in vitro proliferation analyses: (**A**) Temperature rise obtained after application of an alternating magnetic field (385 kHz; 28 kA/m) during 25 min for MNPs coated with PEG400, PEG6000, and βCD at a concentration of 0.5 mg/mL of Fe. The data were represented as the mean of 3 measurements ± standard deviation; (**B**) In vitro proliferation assay of MNPs coated with PEG400, PEG6000, and βCD at 72 h of exposition. Graphs show the percentage of proliferation of T84 at doses ranging from 1–100 µg/mL of Fe_3_O_4_. Results were expressed as mean ± SD of triplicate cultures.

The ability to generate hyperthermia was analyzed after 25 min of application of a high frequency alternating magnetic field. The heating curves obtained are represented in Figure 6A. MNPs coated with PEG400, PEG6000, and βCD were able to increase temperature up to 5.80, 9.27, and 6.03 °C, leading to SAR values of 33.72 ± 0.85, 51.87 ± 2.23, and 33.65 ± 2.63 W/g, respectively. SAR is a relevant measure to characterize the magnetic capabilities of a nanoformulation since it refers to the amount of energy converted into heat in the units of time and mass. This ability to produce heat makes it possible to use nanoformulations as hyperthermia generation agents, understood as the ability to generate a temperature increase when an external magnetic field is applied [41]. This characteristic has been used as a treatment for a wide range of cancers, with very promising results both in vitro and in vivo [42,43]. In this regard, a high SAR value would allow an increase in the capacity to generate heat and therefore decrease the dose of nanoformulation necessary to produce cell death. Some parameters that influence the SAR value are the size and shape of the nanoparticle, the magnetic anisotropy, and the frequency and amplitude of the magnetic field, among others [44]. In this case, @Fe_3_O_4_-PEG6000 presents a higher temperature rise than the rest of the nanoformulations, so it could be the one with the best systems for cellular hyperthermia assays.

To determine the biocompatibility of the different fabricated nanoformulations, a toxicity study was performed. The in vitro antiproliferative effect of all the nanoformulations was analyzed in the human colon cancer cell line T84. SRB assay showed relative proliferation percentages close to 100% for every nanoformulation, with no significant differences compared to control at most doses administered (Figure 6B), showing good in vitro biocompatibility.

Finally, to examine the colloidal stability of the ferrite samples, and to know the particle durability, magnetic ferrite nanospheres were dispersed through sonication in doubly distilled water, phosphate buffered saline (PBS, pH 7.5 or 6.6), and in citrate buffer (pH 3.5) solutions to reach the MNPs concentrations shown in Table 1. The dispersions were kept at 21 °C and protected from light for 2 weeks. In all cases, the colloidal suspensions remained unchanged after this period, demonstrating that they can be well dispersed in aqueous conditions. Therefore, with appropriate surface modifications, these nanospheres may be suitable for clinical diagnostics and drug, protein, virus, or bacteria transport.

## 4. Conclusions

We successfully carried out the synthesis of MNPs with several functionalized polymeric hydrophilic coatings in a single-step reaction following the solvothermal method. To the best of our knowledge, no other study has documented the use of this method to obtain magnetic spheres with a functionalized polymeric coating. The nanoparticles had diameters in the range of 90 to 250 nm, and each magnetic core composing the cluster had a size of about 10 nm, which was concordance with the calculated Scherrer equation values (8.7 ± 0.4 nm).

Two of these coatings (PEG400-N_3_ and PEG400-Pro) allow the performance of click chemistry reactions, facilitating the construction of more complex structures from basic units. This fact was illustrated in the case of the cannabidiol-propargyl derivative (CBD-Pro), which was covalently attached to the MNPs functionalized surface (@Fe_3_O_4_-PEG400-N_3_) to obtain @Fe_3_O_4_-PEG400-CBD systems.

The magnetic properties of PEG- and βCD-coated Fe_3_O_4_ systems were investigated at room temperature, and SPM behavior was confirmed, with maximum saturation mass magnetization reaching values of about 60 emu/g for PEG6000 systems. Coercive force values have been found to be close to zero since magnetization curves do not show any hysteresis, this fact being in concordance with the SPM behavior of the nanosystems. MNPs were also analyzed as heat generation agents, producing an optimum temperature rise of about 5–10 °C, and SAR values after 25 min of being subjected to a high frequency alternating magnetic field, showing their possible use as hyperthermia generation agents. This fact, together with the great biocompatibility results found in the T84 human colon cancer cell line, and their ability to form stable suspensions in PBS and citrate buffers, opens a wide range of applications for prepared MNPs within biomedicine.

## Figures and Tables

**Table 1:** Sizes and concentrations of MNPs with different polymeric coatings.

MNP	Size (nm)	MNPs Concentration (mg/mL)	Fe_3_O_4_ Concentration (mg/mL)
@Fe_3_O_4_-PEG400	250	6.3	0.2
@Fe_3_O_4_-PEG6000	150	7.5	0.4
@Fe_3_O_4_-PEG400-N_3_	90	6.7	0.3
@Fe_3_O_4_-PEG400-Pro	200	6.9	0.3
@Fe_3_O_4_-βCD	155	7.1	0.4

## Data Availability

Data supporting reported results can be found at the Department of Organic Chemistry (University of Málaga).

## Supplementary Materials

The following supporting information can be downloaded at: https://www.mdpi.com/article/10.3390/pharmaceutics16091226/s1, Figure S1: ^1^H-NMR spectrum (500 MHz, CDCl_3_) of PEG400-Br; Figure S2: ^13^C-NMR spectrum (100 MHz, CDCl_3_) of PEG400-Br; Figure S3: FT-IR spectra of PEG400 (blue line) and PEG400-Br (black line); Figure S4: ^1^H-NMR spectrum (500 MHz, CDCl_3_) of PEG400-N_3_; Figure S5: ^13^C-NMR spectrum (100 MHz, CDCl_3_) of PEG400-N_3_; Figure S6: FT-IR spectrum of PEG400-N_3_; Figure S7: ^1^H-NMR spectrum (500 MHz, CDCl_3_) of PEG400-Pro; Figure S8: FT-IR spectrum of PEG400-Pro; Figure S9: MALDI-TOF-MS spectrum of PEG400 with TFA matrix and Na^+^ ionization agent; Figure S10: MALDI-TOF-MS spectrum of PEG400-N_3_ with TFA matrix and Na^+^ ionization agent; Figure S11: MALDI-TOF-MS spectrum of PEG400-Pro with TFA matrix and Na^+^ ionization agent; Figure S12: ^1^H-NMR spectrum (500 MHz, CDCl_3_) of CBD; Figure S13: ^1^H-NMR spectrum (500 MHz, CDCl_3_) of CBD-Pro-1; Figure S14: ^1^H-NMR spectrum (500 MHz, CDCl_3_) of CBD-Pro-2; Figure S15: FT-IR spectrum of @Fe_3_O_4_-PEG400-CBD.
