# Supplementary material for: Magnetic Nanoparticles with On-Site Azide and Alkyne Functionalized Polymer Coating in a Single Step through a Solvothermal Process
Source: Pharmaceutics. 2024 Sep 19;16(9):1226. doi: 10.3390/pharmaceutics16091226 (PMC11435388; doi:10.3390/pharmaceutics16091226)
Supplement: Supplementary file 1 [file pharmaceutics-16-01226-s001.zip › pharmaceutics-3151272-supplementary.pdf]

Magnetic nanoparticles with on-site azide and alkyne functionalized polymer coating in a single step through a solvothermal process

Romualdo Mora-Cabello,[1] David Fuentes-Ríos,[1] Lidia Gago,[2,3,4] Laura Cabeza,[2,3,4] Ana Moscoso,[1] Consolación Melguizo,[2,3,4] José Prados,[2,3,4] Francisco Sarabia,[1] and Juan Manuel López-Romero[1,*]

1. Department of Organic Chemistry, Faculty of Sciences, University of Málaga, 29071 Málaga, Spain

2. Institute of Biopathology and Regenerative Medicine (IBIMER), Biomedical Research Center (CIBM), 18100 Granada, Spain

3. Instituto de Investigación Biosanitaria de Granada (Ibs.GRANADA), 18012 Granada, Spain

4. Department of Anatomy and Embryology, University of Granada, 18071 Granada, Spain.

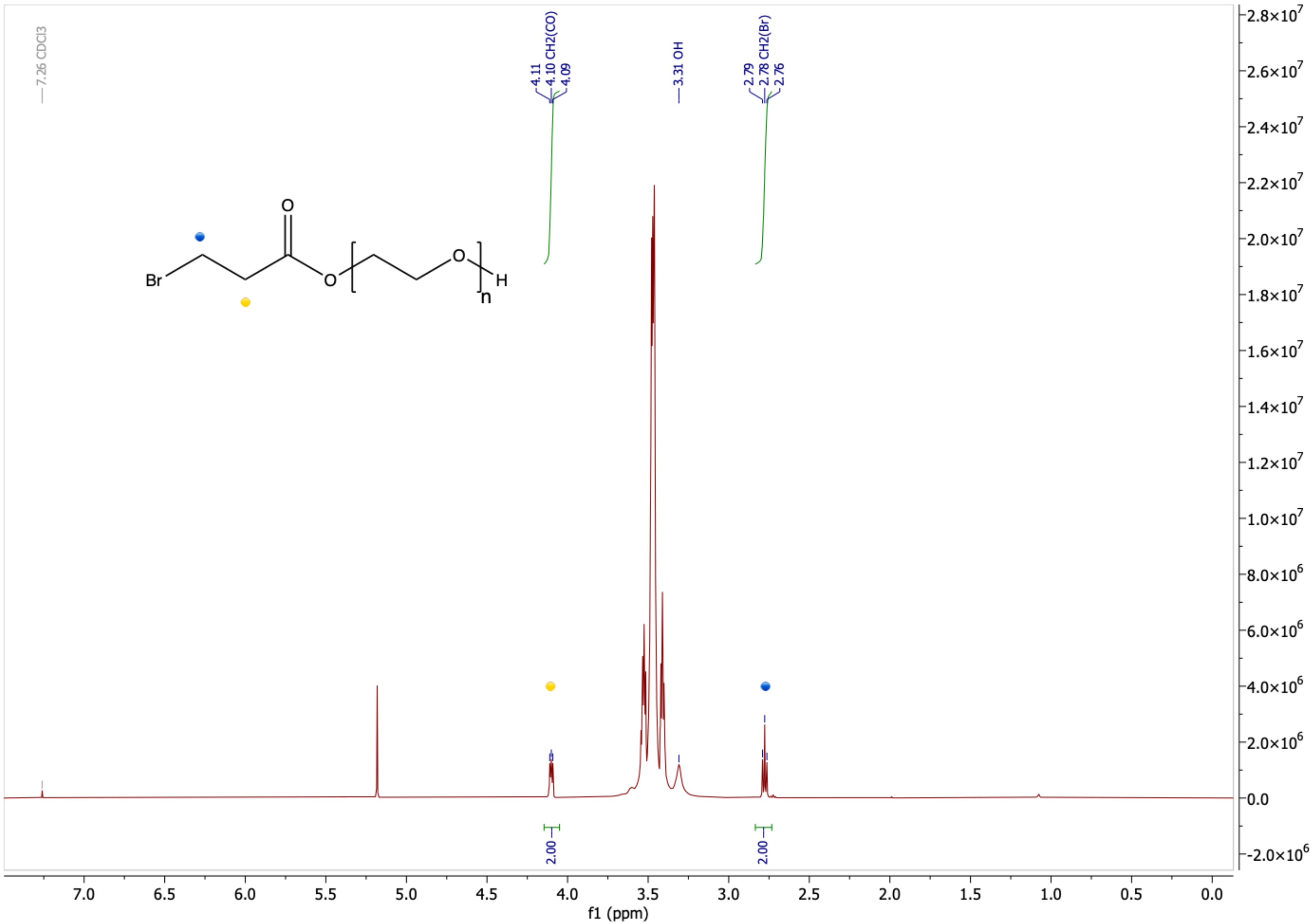

Figure S1. ^{1}H-NMR spectrum (500 MHz, $CDCl_3$) of PEG400-Br

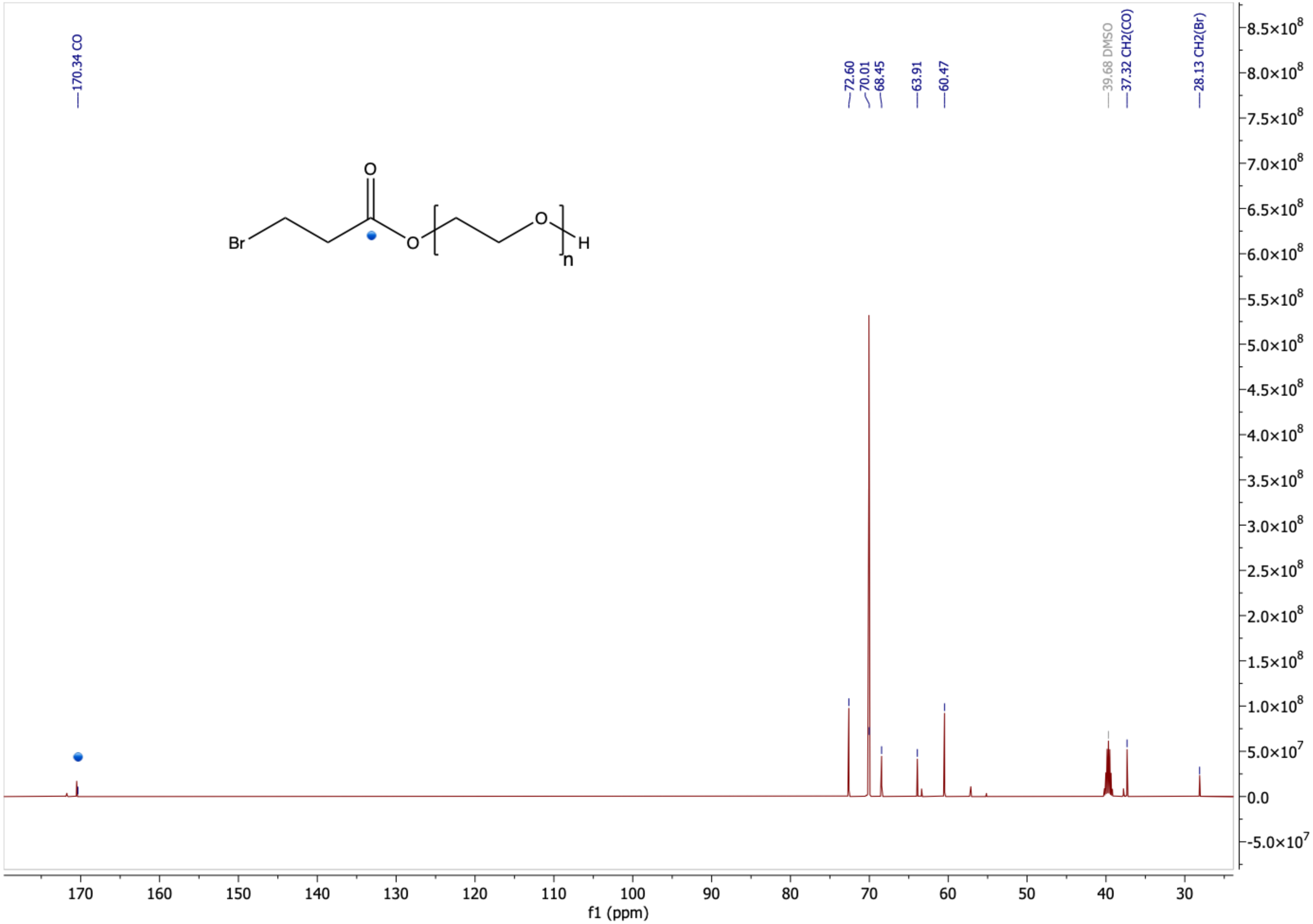

Figure S2. ^{13}C-NMR spectrum (100 MHz, $CDCl_3$) of PEG400-Br

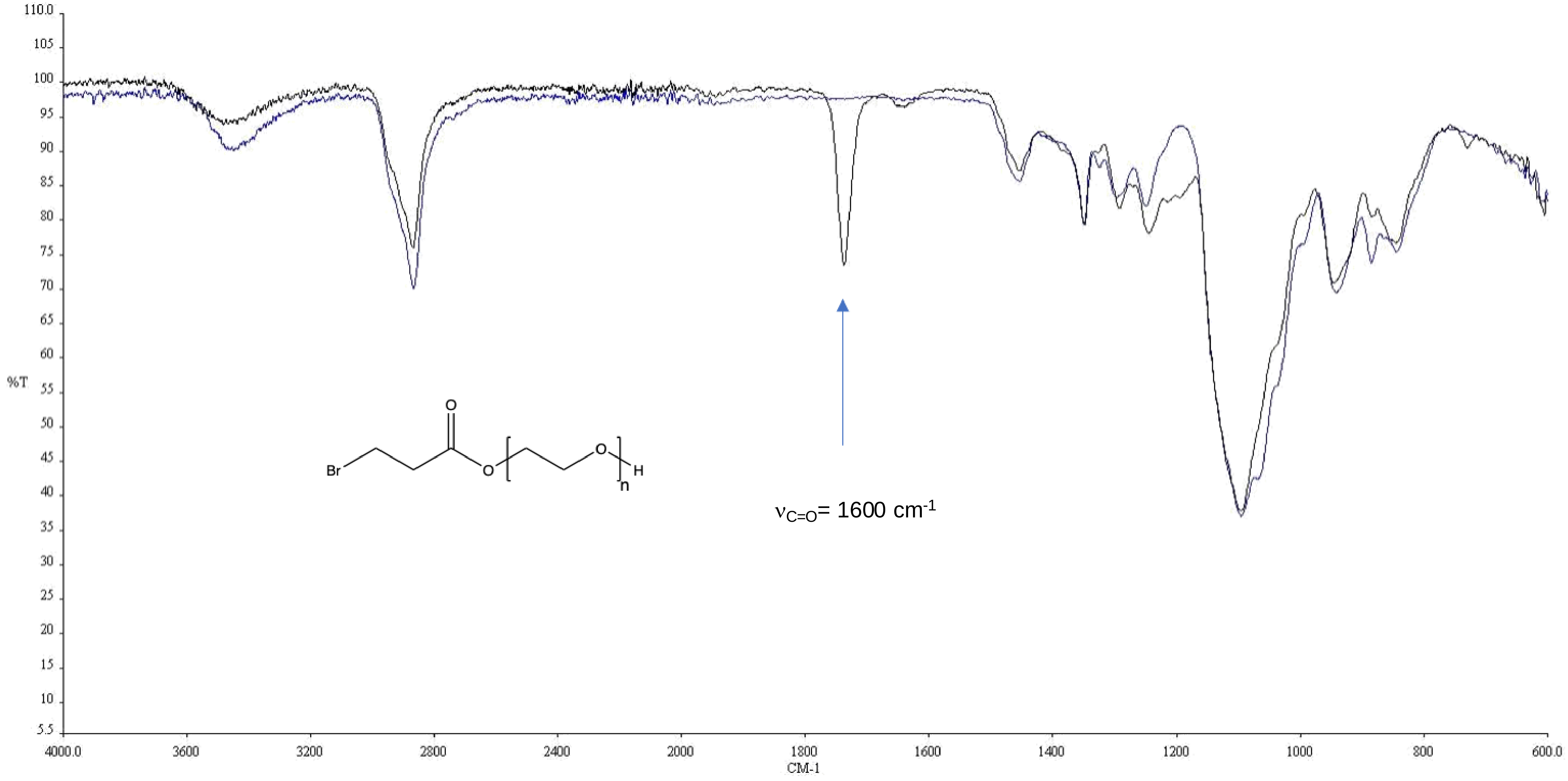

Figure S3. FT-IR spectra of PEG400 (blue line) and PEG400-Br (black line)

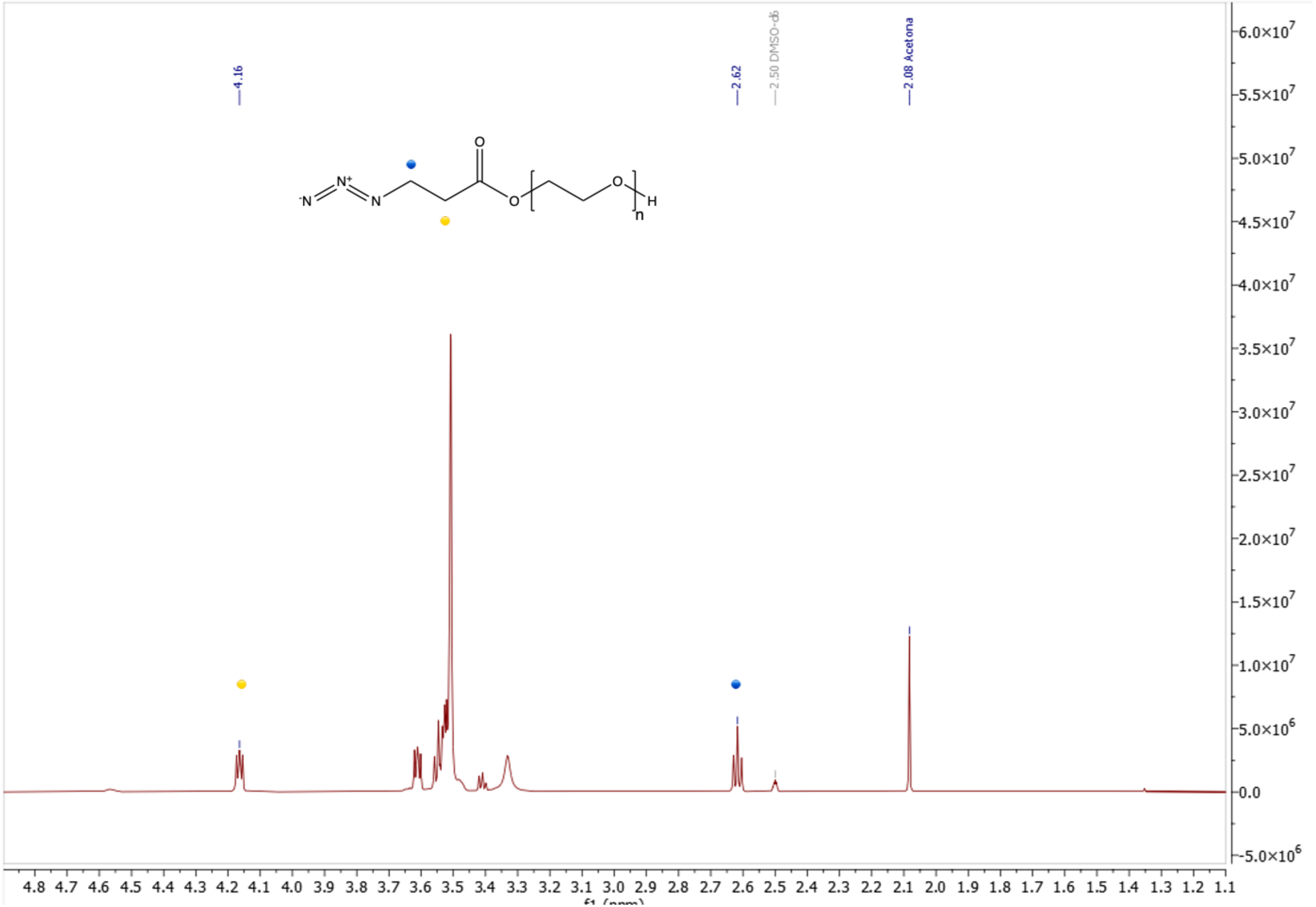

Figure S4. ^{1}H-NMR spectrum (500 MHz, $CDCl_3$) of PEG400-N_3

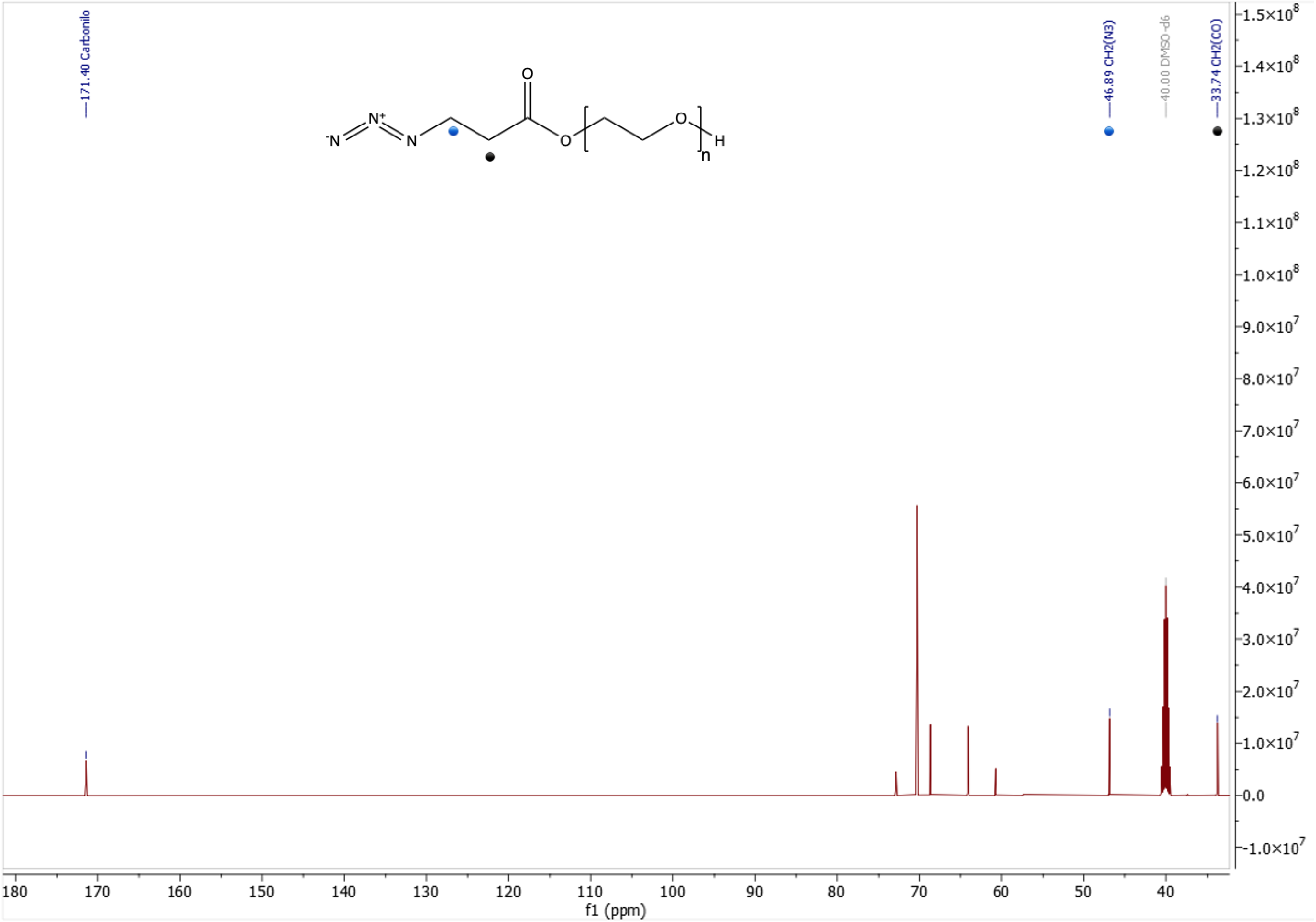

Figure S5. ^{13}C-NMR spectrum (100 MHz, $CDCl_3$) of PEG400-N_3

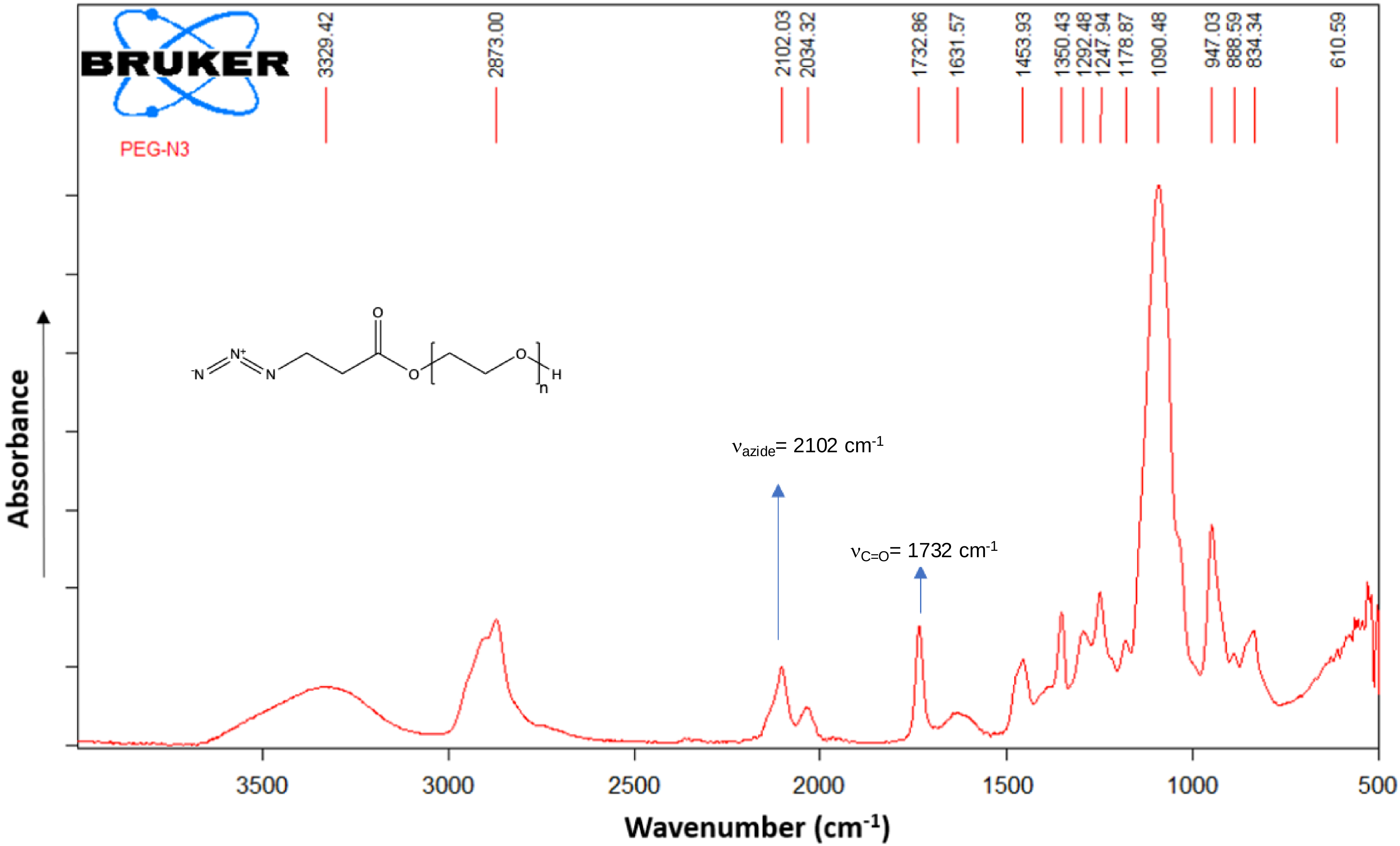

Figure S6. FT-IR spectrum of PEG400-N_3

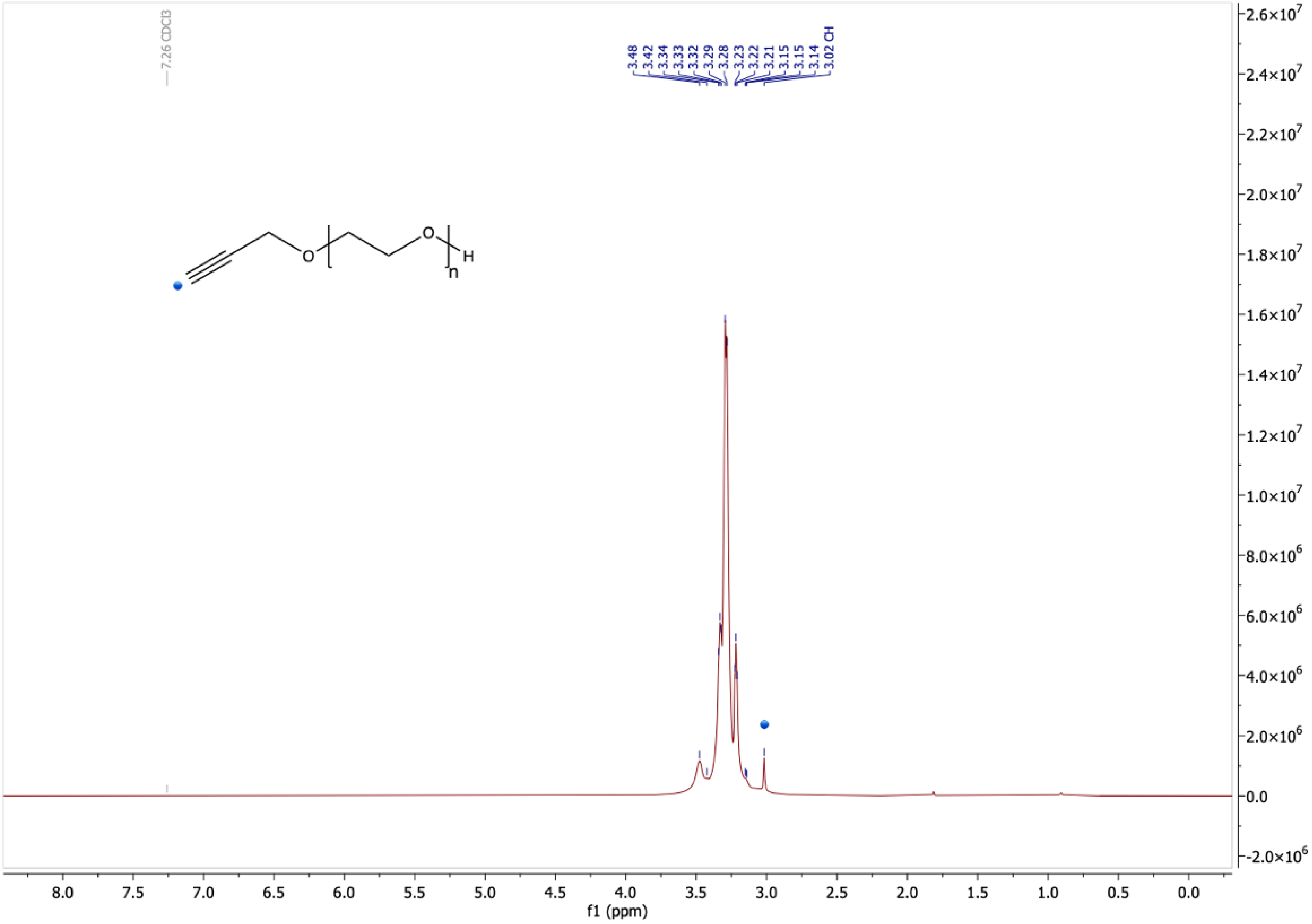

Figure S7. ^{1}H-NMR spectrum (500 MHz, $CDCl_3$) of PEG400-Pro

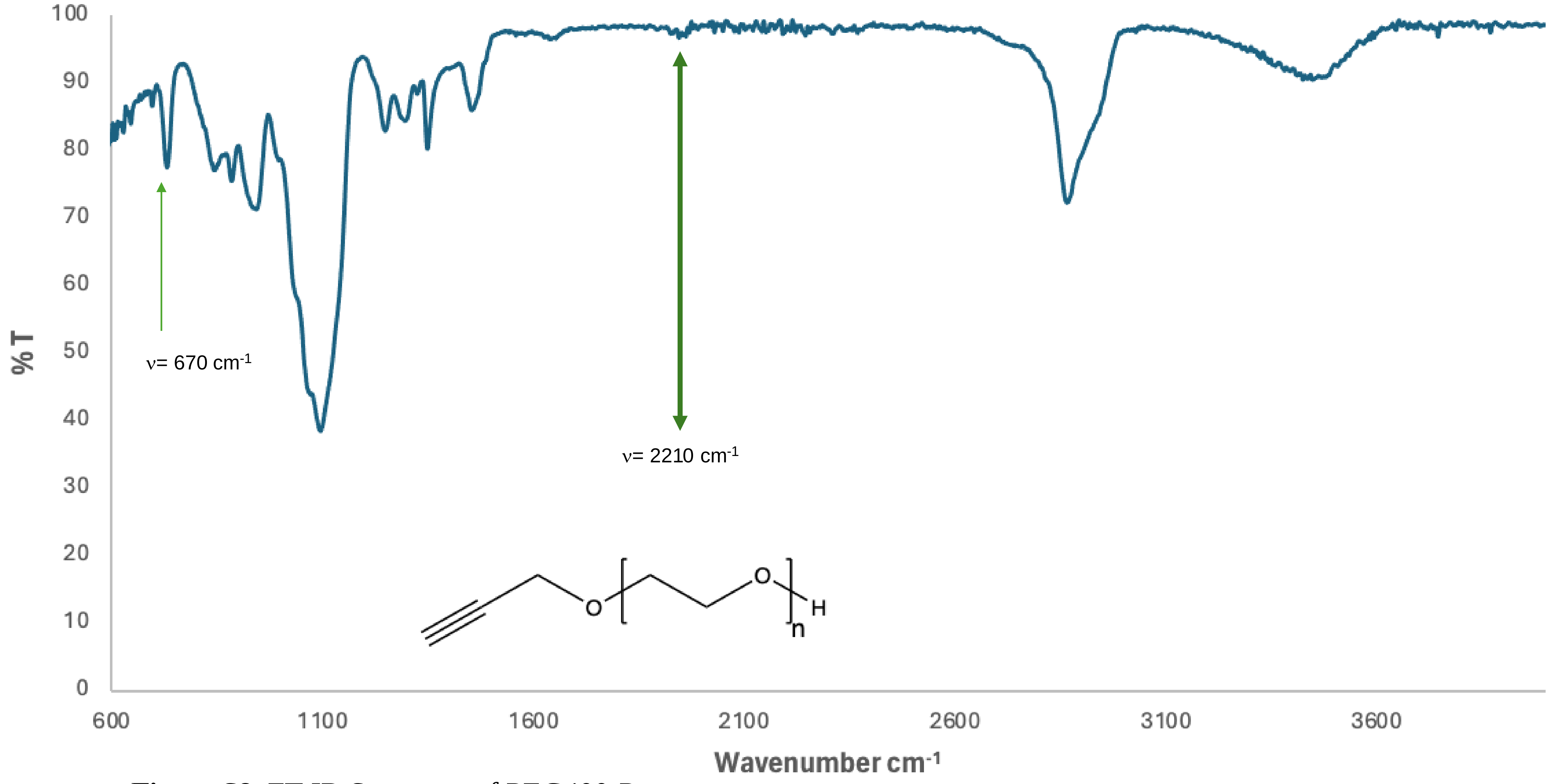

Figure S8. FT-IR Spectrum of PEG400-Pro

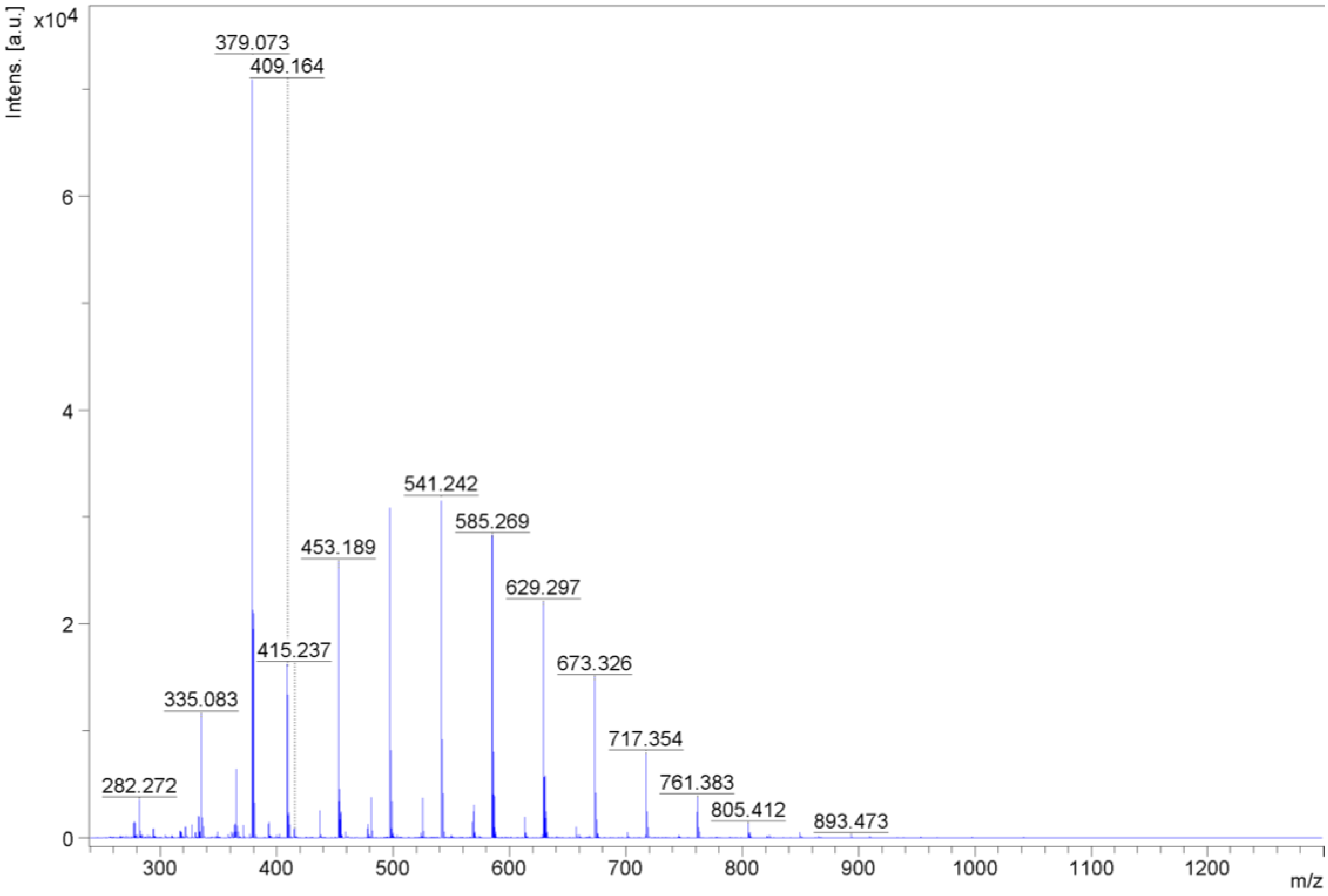

Figure S9. MALDI-TOF-MS spectrum of PEG400 with TFA matrix and Na^{+} ionization agent

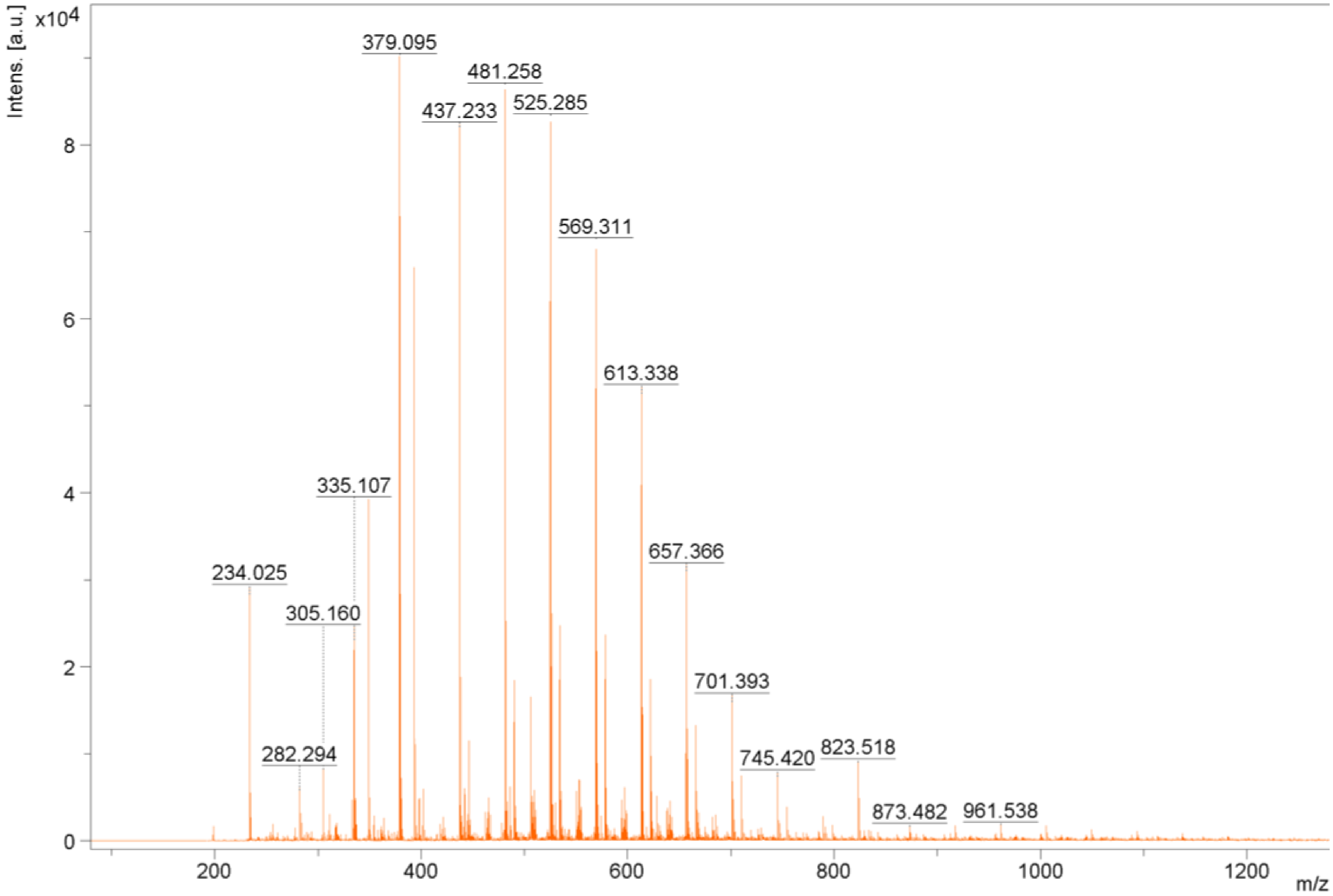

Figure S10. MALDI-TOF-MS spectrum of PEG400-N_3 with TFA matrix and Na^+ ionization agent

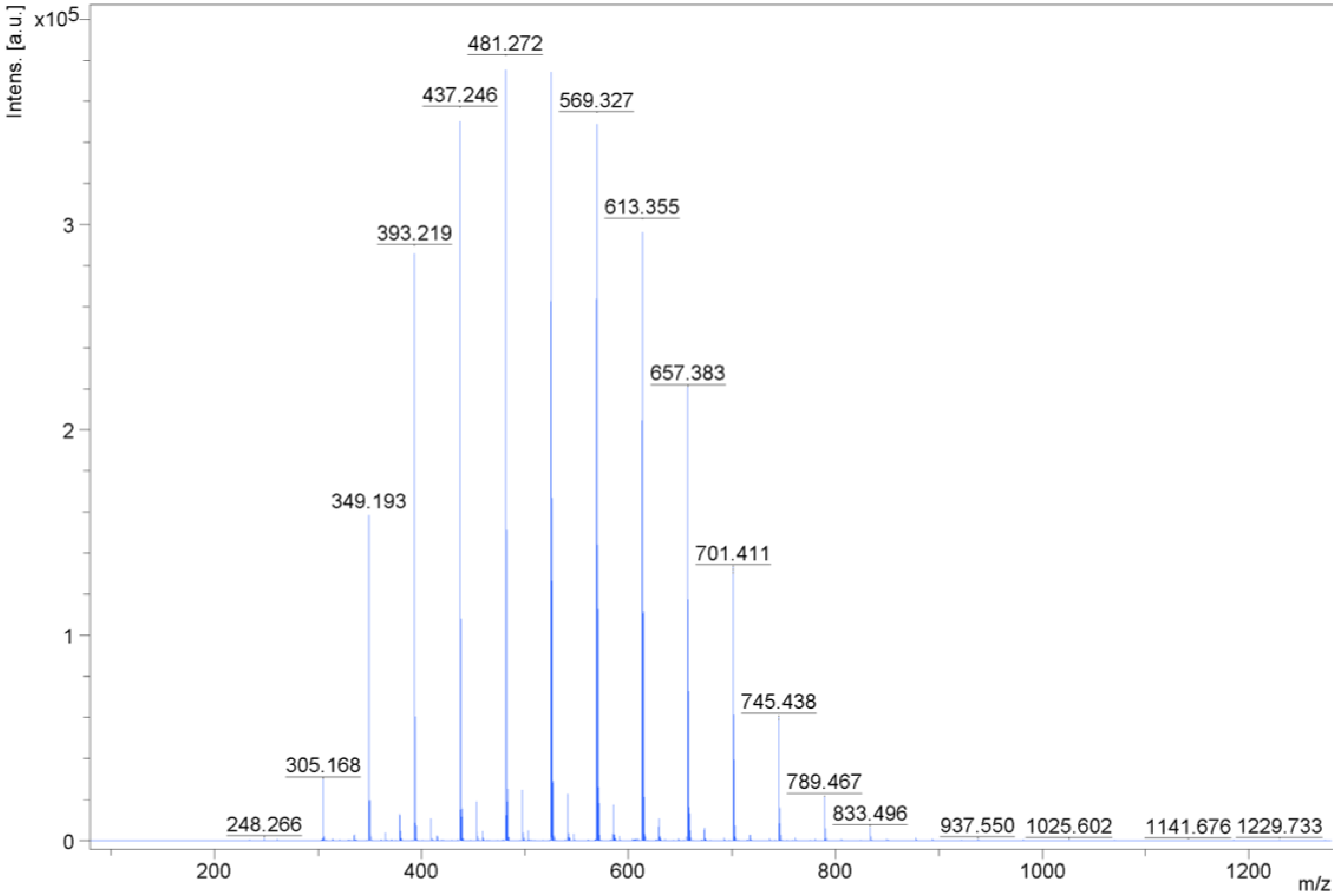

Figure S11. MALDI-TOF-MS spectrum of PEG400-Pro with TFA matrix and Na^+ ionization agent

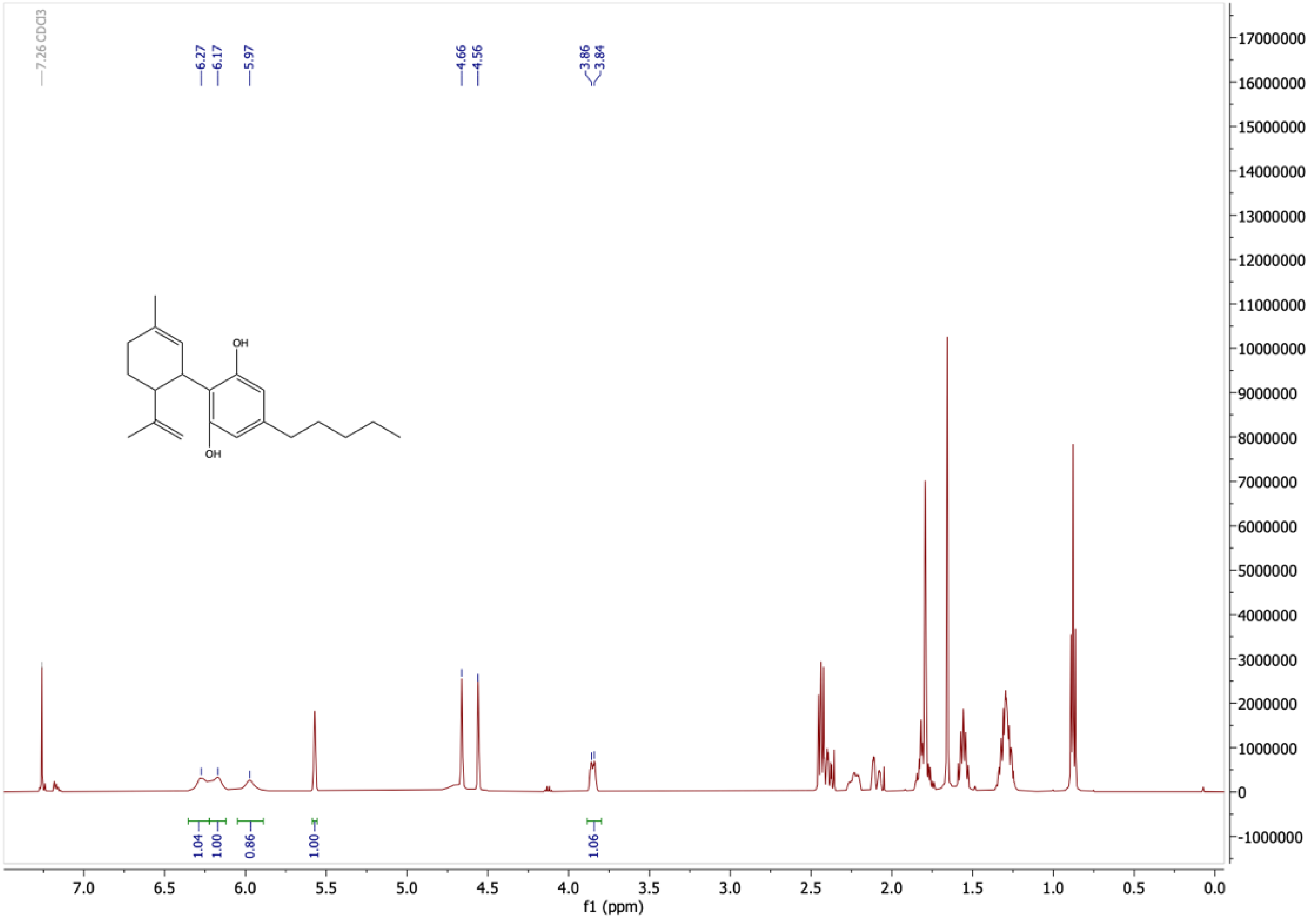

Figure S12. ^{1}H-NMR spectrum (500 MHz, $CDCl_3$) of CBD

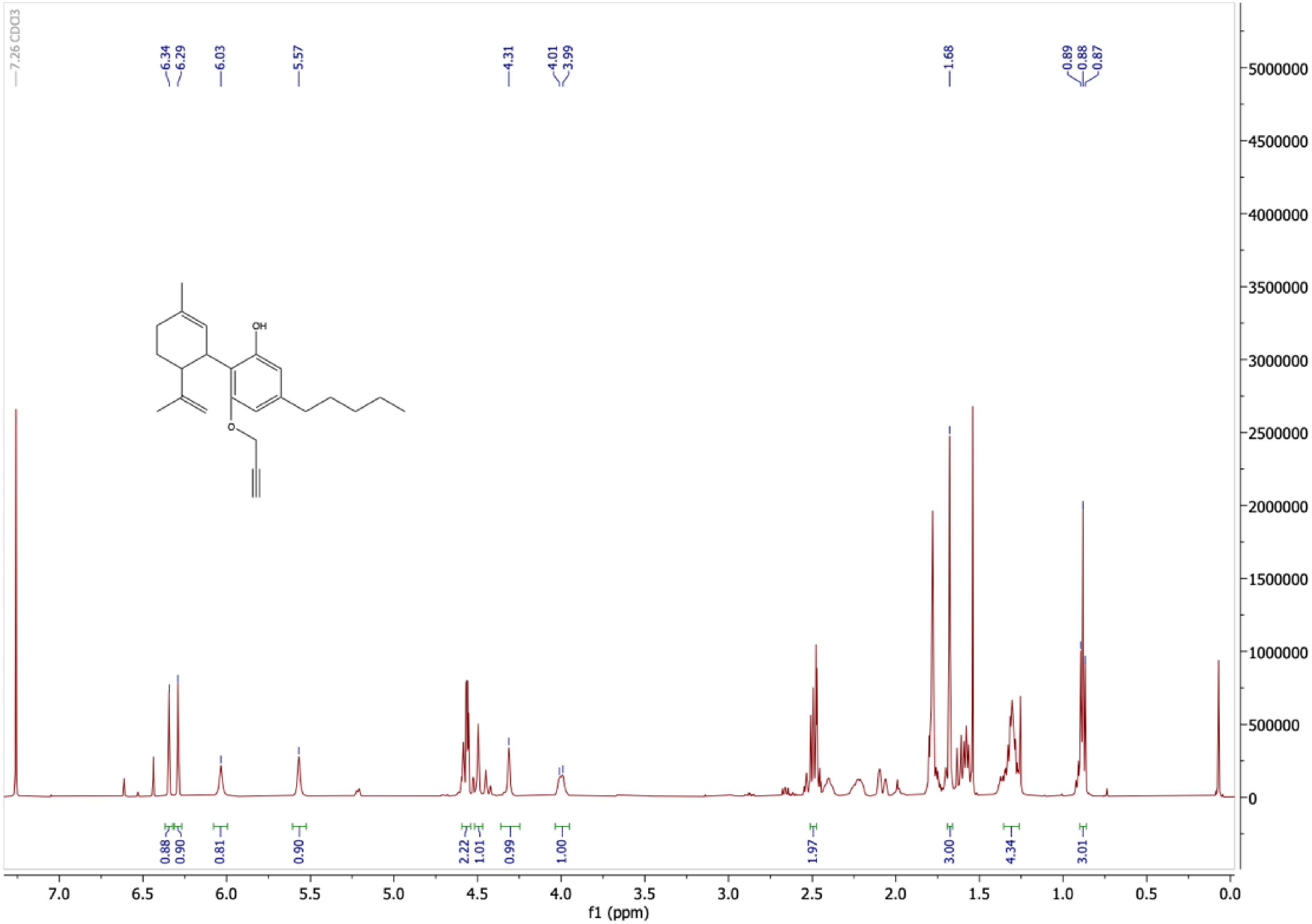

Figure S13. ^{1}H-NMR spectrum (500 MHz, $CDCl_3$) of CBD-Pro-1

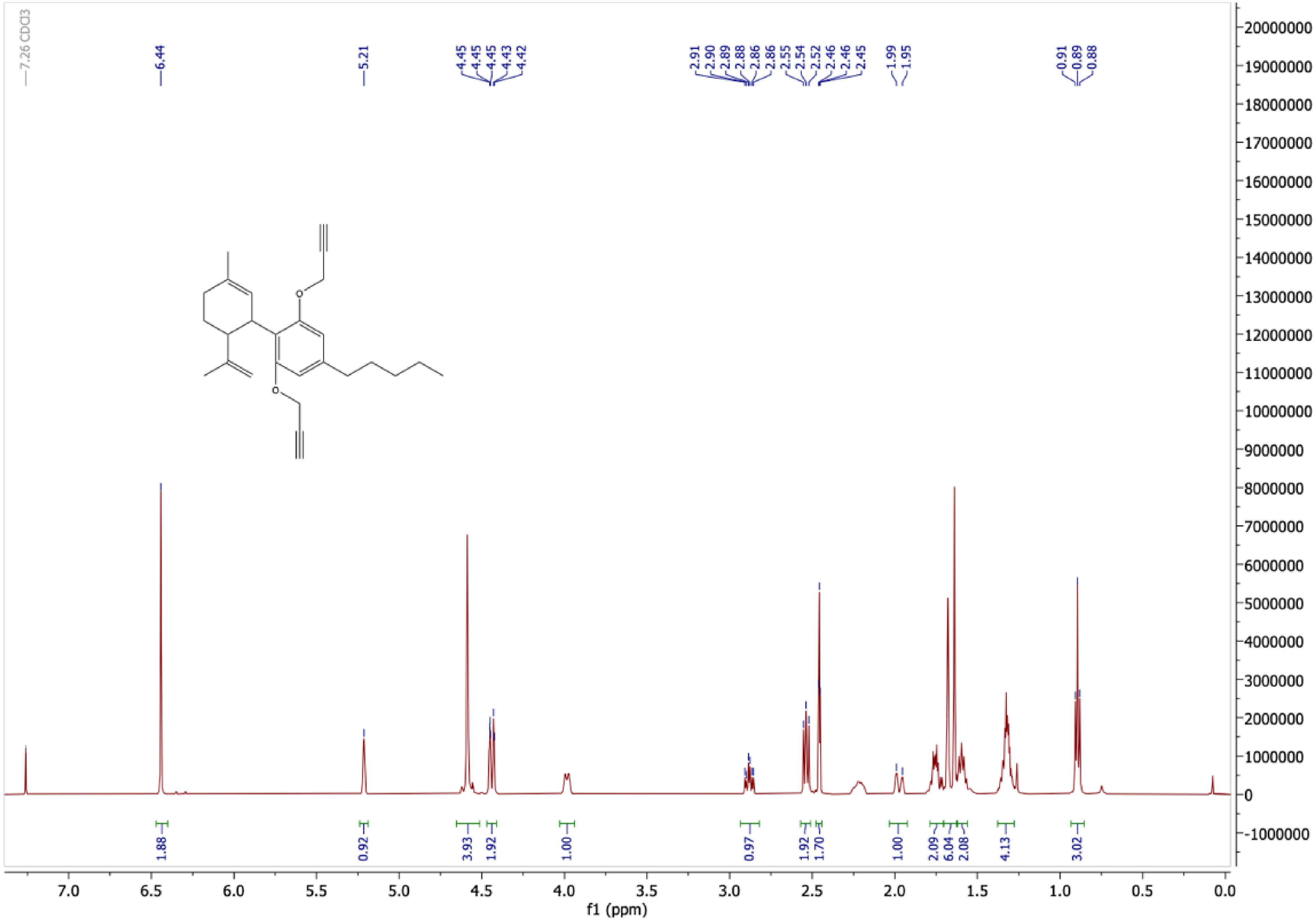

Figure S14. ^{1}H-NMR spectrum (500 MHz, $CDCl_3$) of CBD-Pro-2

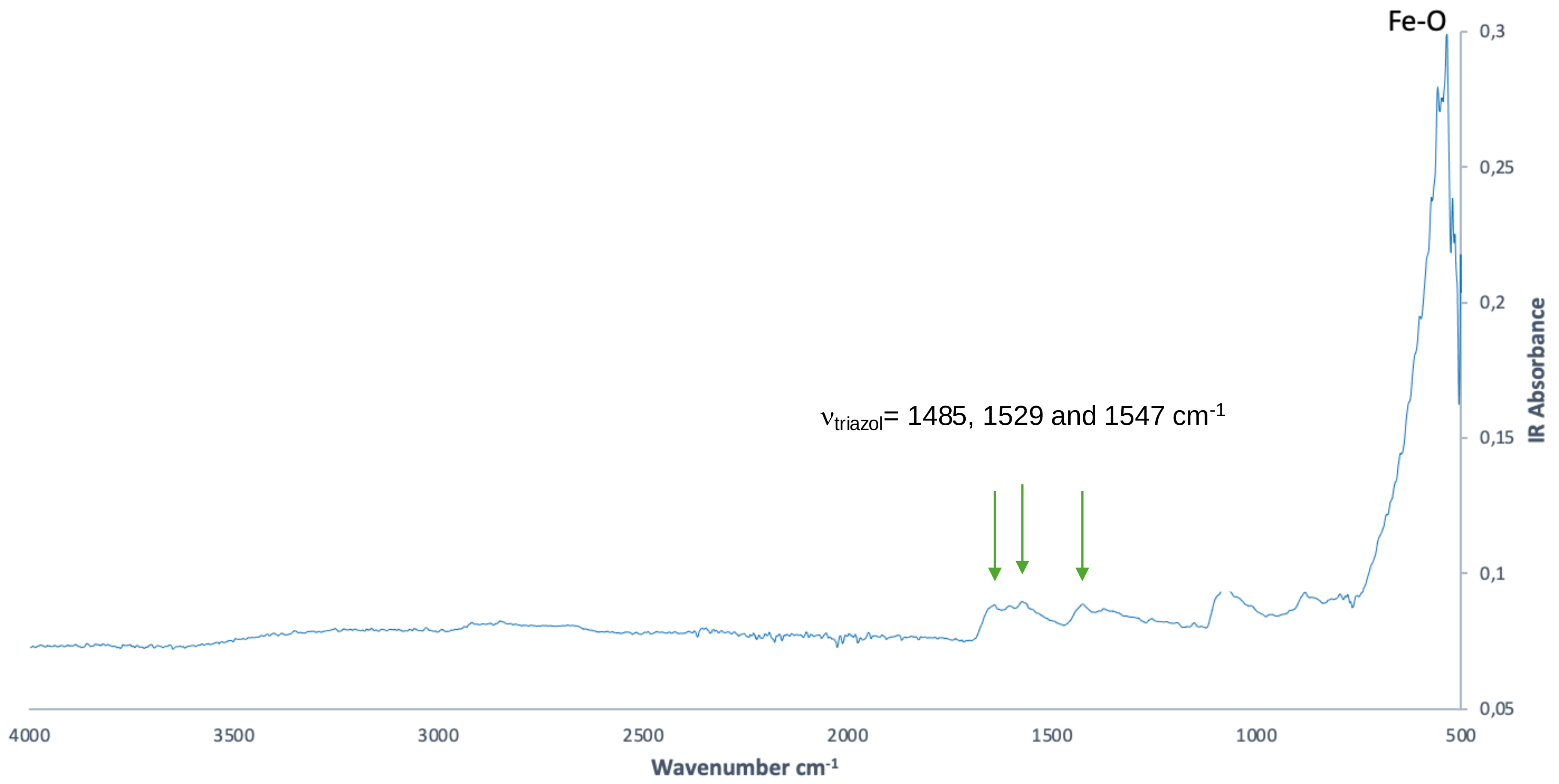

Figure S15. FT-IR spectrum of @Fe_3O_4-PEG400-CBD
